# ADT-OH exhibits anti-metastatic activity on triple-negative breast cancer by combinatorial targeting of autophagy and mitochondrial fission

**DOI:** 10.1038/s41419-024-06829-w

**Published:** 2024-06-28

**Authors:** Shihui Yu, Zhiting Cao, Fangfang Cai, Yingying Yao, Xiaoyao Chang, Xiaoyang Wang, Hongqin Zhuang, Zi-Chun Hua

**Affiliations:** 1 The State Key Laboratory of Pharmaceutical Biotechnology, College of Life Sciences, Nanjing University, Nanjing, P. R. China; 2 School of Biopharmacy, China Pharmaceutical University, Nanjing, 211198 China; 3 Changzhou High-Tech Research Institute of Nanjing University and Jiangsu TargetPharma Laboratories Inc., Changzhou, 213164 P. R. China

**Keywords:** Breast cancer, Mitophagy, Breast cancer, Cell invasion

## Abstract

High basal autophagy and enhanced mitochondrial fission in triple-negative breast cancer (TNBC) cells support cell migration and promote plasticity of cancer cell metabolism. Here, we suggest a novel combination therapy approach for the treatment of TNBC that targets Drp1-mediated mitochondrial fission and autophagy pathways. Hydrogen sulfide (H_2_S) mediates a myriad of biological processes, including autophagy and mitochondrial function. In this study, we demonstrated that 5-(4-hydroxyphenyl)-3H-1,2-dithiole-3-thione (ADT-OH), one of the most widely utilized sustained-release H_2_S donors, effectively suppresses metastasis of TNBC cells in the absence of proliferation inhibition in vitro and in vivo. ADT-OH treatment ameliorated autophagy flux by suppressing autophagosome formation and induced mitochondrial elongation through decreasing expression of dynamin-related protein 1 (Drp1) and increasing expression of mitochondrial fusion protein (Mfn2). At the same time, ADT-OH downregulated mitophagy flux and inhibited mitochondrial function, eventually leading to the inhibition of migration and invasion in TNBC cells. In vivo, intraperitoneal administration of ADT-OH revealed a potent anti-metastatic activity in three different animal models, the MDA-MB-231 orthotopic xenograft model, the 4T1-Luci orthotopic model and the 4T1-Luci tail vein metastasis model. However, ADT-OH has an extremely low water solubility, which is a significant barrier to its effectiveness. Thus, we demonstrated that the solubility of ADT-OH in water can be improved significantly by absorption with hydroxypropyl-β-cyclodextrin (CD). Remarkably, the obtained CD-ADT-OH demonstrated superior anti-cancer effect to ADT-OH in vivo. Altogether, this study describes a novel regulator of mammalian mitochondrial fission and autophagy, with potential utility as an experimental therapeutic agent for metastatic TNBC.

## Introduction

According to GLOBOCAN 2020, the proportion of patients diagnosed with breast cancer has surpassed lung cancer as the most common malignancy [1]. Among all newly diagnosed breast cancers, triple-negative breast cancer (TNBC) which causes the highest mortality rate among all breast cancers represents approximately 15–20% [2, 3]. Due to the molecular heterogeneity of TNBC and lack of well-defined molecular targets, targeted therapy for the most aggressive breast cancer subtype, TNBC, remains limited and conventional chemotherapy remains the standard of care [4]. However, there is no standard chemotherapy regimen for patients with metastatic TNBC [5]. Therefore, novel targeted therapeutics for TNBC, especially metastatic TNBC, are urgently needed.

Metabolic reprogramming, a recognized hallmark of cancer, endows cancer cells with adaption in inhospitable microenvironment, including the stress environment generated by anticancer therapies [6]. Among these metabolic adaptations, cancer cells, including TNBC cells, use glycolysis as well as mitochondrial oxidation to produce ATP and autophagy or other specific forms of autophagy, such as mitophagy, to recycle cellular components [7]. Autophagy can be activated as a pro-survival pathway and a resistance mechanism against chemotherapeutic agents in TNBC cells [8]. Therefore, inhibition of autophagy has been considered to be a promising strategy for TNBC treatment. However, autophagy inhibition alone is insufficient for TNBC treatment, which is usually regarded as the adjuvant strategy for improving chemotherapy efficiency in TNBC preclinical models [9, 10]. To address the limited efficacy of autophagy inhibition in TNBC preclinical models, it has been further proposed that combinatorial targeting of autophagy and mitochondria function, rather than autophagy inhibition alone, would be an effective treatment for basal-like breast cancers [11].

Migratory properties of cancer cells depend on mitochondrial function [12]. Mitochondria are highly dynamic, constantly changing their morphology and location by a balance between active fusion and fission to accommodate functional changes [13, 14]. Among them, mitochondrial fission and fusion are regulated by large GTPases such as dynamin-related protein 1 (Drp1) and mitofusins (Mfns), respectively [15]. Evidence suggests that high levels of mitochondrial fission activity have been found to be associated with high metastasis and invasiveness in some cancer cells, including invasive breast cancer cells [16, 17]. Compared to non-invasive cells, the expression levels of Drp1 are up-regulated while Mfns are down-regulated in invasive breast cancer cells. Silencing Drp1 or overexpression of Mfn1 in invasive breast cancer cells could induce mitochondrial elongation and inhibit cell metastasis [18]. Thus, we further propose that combinatorial targeting of autophagy and mitochondrial fission mediated by Drp1 may be an effective treatment option for TNBC.

Hydrogen sulfide (H_2_S) has emerged as the third major gasotransmitter after carbon monoxide (CO) and nitric oxide (NO) that mediates a myriad of biological processes, including autophagy [19] and mitochondrial function [20]. Recently, an increasing amount of evidence suggests that endogenously produced or exogenously administered H_2_S could exhibit anti-autophagy effect by modulating numerous signaling pathways, such as PI3K/AKT/mTOR, Nrf2-ROS-AMPK, AMPK/mTOR and other signaling pathways [19]. H_2_S has also been reported to regulate cell mitochondrial functions and metabolism. For example, 5-(4-hydroxyphenyl)-3H-1,2-dithiocyclopentene-3-thione (also named as ADT-OH), a sustained-release H_2_S donor, have been proved to induce mitochondrial uncoupling through SQR [21]. Based on above evidences, combinatorial targeting of autophagy and mitochondria function by H_2_S donor appears to be feasible. Additionally, our previous study demonstrated that ADT-OH has effective therapeutic benefits in malignant melanoma [22, 23]. Furthermore, Dong et al. connected Hyaluronic acid (HA) with ADT-OH through chemical reactions and found that HA-ADT suppressed the PI3K/AKT/mTOR and RAS/RAF/MEK/ERK signaling pathways, thereby inhibiting the proliferation, migration, and invasion of human breast cancer cells [24]. Therefore, ADT-OH may be a preferable option for the treatment of TNBC given its strong therapeutic potential. However, ADT-OH shows low permeability and aqueous solubility, necessitating substantial doses for in vivo delivery. Cyclodextrin, especially hydroxypropyl-β-cyclodextrin (CD), is crucial for increasing the permeability and solubility of the anticancer drugs [25]. CD have a unique ring structure with a hydrophilic outer surface and an internal hydrophobic cavity. Cyclodextrins create non-covalent inclusion complexes when they interact with molecules of the suitable size. This inclusion complex has increased solubility and stability compared to hydrophobic medications. Here, we tried to use β-cyclodextrin as a supramolecular drug reservoir to deliver ADT-OH. We therefore hypothesized that covalent cross-linking of CD would enable the formation of cyclodextrin-ADT-OH particles (CD-ADT-OH) with high solubility and therapeutic effect.

In this study, we used highly metastatic TNBC cell lines, MDA-MB-231 and 4T1, and non-metastatic breast cancer cell, MCF-7, which exhibit different basial autophagy and mitochondrial fission activity. The use of cell lines with different invasive properties allowed us to better visualize the differences in the effects of ADT-OH. By contrasting the different effects of ADT-OH on TNBC cells and non-TNBC cells, we unveiled that the suppression of mitochondrial fission and autophagy flux contributes to decrease in mitophagy flux and mitochondrial function, leading to the inhibition of migration and invasion without affecting proliferation in TNBC cells. We further demonstrated that ADT-OH could significantly reduce metastatic capacity in vivo using three different TNBC models, MDA-MB-231 orthotopic xenograft model, 4T1-Luci orthotopic model and 4T1-Luci tail vein metastasis model. More importantly, we demonstrated that the CD-ADT-OH exhibited better efficacy than ADT-OH alone in inhibiting tumor metastasis in vivo. Overall, our study demonstrated the potential therapeutic utility of combinatorial targeting of autophagy and mitochondrial fission by ADT-OH for metastatic TNBC.

## Materials and methods

### Cell lines

Human breast cancer cell lines MDA-MB-231, MCF-7 and 4T1-luci were purchased from American Type Culture Collection (ATCC, USA) or maintained in our laboratory and cultured in Dulbecco’s modified Eagle’s medium (DMEM; Sangon, China) supplemented with 10% fetal bovine serum (FBS; Hyclone, USA; SV30087.03), 100 IU/ml penicillin, and 100 μg/ml streptomycin (Invitrogen, USA). The stably mouse breast cancer cell line 4T1-luci cell line was cultured in RPMI 1640 medium (Sangon, China) supplemented with 10% fetal bovine serum, penicillin (100 IU/ml) and streptomycin (100 μg/ml). Cells were grown in an incubator with a humidified atmosphere of 95% air and 5% CO_2_ at 37 °C. The sample size for cellular experiments was 3 or 5 per group.

### Drug formulations

5-(4-hydroxyphenyl)-3H-1, 2-dithiole-3-thione (ADT-OH) was synthesized by Suzhou University. In order to prepare CD-ADT-OH, 2.5 mg CD was dissolved in 5 ml PBS and ADT-OH (100 mg/ml, DMSO) was added gradually while stirring. For in vitro studies, ADT-OH was prepared in dimethyl sulfoxide (DMSO) (100 μM stock) and diluted to their final concentrations in cell culture medium prior to in vitro assays, whereas for in vivo studies, the drug was dissolved in 0.5% (w/v) methylcellulose solution.

### Cell proliferation assay

Cell proliferation assay was performed using the Cell Counting Kit-8 kit (CCK-8; Beyotime Biotech, China). Cells were seeded at 5000 cells per well in a 96-well plate and incubated with ADT-OH (0–200 μM) for 24 h or 48 h before 10 μl of CCK-8 was added. The absorbance at 450 nm was measured after 2 h of incubation using a microplate spectrophotometer (model MCC/ 340; Titertek Instruments Inc, USA). IC50 values were calculated by plotting these data as the log10 drug concentration (nM) versus the cell viability and fitting to a dose-response curve. Curve fitting was performed with GraphPad Prism 8.0 Software.

### Cell migration assay

The effect of ADT-OH on breast cancer cell migration was tested by 2 different techniques: the scratch wound healing assay and the transwell migration assay with a Boyden chamber (8.0 μm pore size; Millipore, USA). Briefly, for the wound healing assay, cells were cultured in 6-well plates until reaching confluence and began to be starved for 12 h before scratching. A sterile P-200 micropipette was used to create a wound in the middle of the confluent cell layer. After washing with PBS to remove detached cells and application of ADT-OH as migration inhibitor, the wound was imaged by an optical microscope (Zeiss Axioplan-2 microscope; Carl Zeiss, Germany) at 0 h and 48 h and the area of the wound was quantified by the ImageJ software (Fiji; NIH, USA). For the transwell migration assay, 5 × 10^4^ cells in 100 μl of serum-free medium containing different concentrations of ADT-OH were seeded in the upper chambers uncoated with Matrigel, while the lower chamber was filled with 750 μl medium containing 10% FBS. After incubation for 24 h or 48 h, cells on the lower membrane surface were fixed in 4% paraformaldehyde, stained with 0.1% crystal violet (Solarbio, China; G1061) for 30 min and photographed (×100) in 5 independent fields for each sample. The number of stained cells per field was counted and calculated by ImageJ software.

### Characterization of CD-ADT-OH

Vibrational spectra were obtained by Fourier transform infrared spectroscopy (FTIR) analysis, in a Bruker TENSOR 27 FTIR spectrometer, performed on KBr pellets in the range of 4000–500 cm^−1^. The UV/vis-absorption analysis was performed in a BioTek Synergy H1 microplate reader, which works in the wavelength range of 220 to 999 nm.

### Flow cytometry analysis of apoptosis and cell cycle

Breast cancer cells were seeded overnight in 12-well plates and then treated with ADT-OH at a concentration of 0, 50, 100 μM for 48 h. Cells were trypsinized and harvested (including supernatant) before being stained with propidium iodide (PI; 20 μg/mL; Sigma, Germany) and fluorescein isothiocyanate (FITC) Annexin V (2 μg/mL) prepared in our laboratory to analyze apoptosis. For cell cycle analysis, the Cell Cycle and Apoptosis Analysis Kit (Beyotime Biotech, China) was applied according to the manufacturer’s instructions. Flow cytometry analysis of cell cycle and apoptosis was performed using NovoCyte flow cytometer (ACEA Biosciences, USA) equipped with the NovoExpress software.

### Confocal microscopy

Confocal microscopy was performed using a Leica TCS SP8 inverted laser scanning confocal microscope (Leica Microsystems, Germany) or a Zeiss LSM710 confocal microscope (Carl Zeiss, Germany), with the preset settings for DAPI (Ex: 350 nm, Em: 415–500 nm), GFP (Ex: 488 nm, Em: 500–550 nm), RFP (Ex: 561 nm, Em: 570–620 nm). Confocal images were processed using Leica LAS AF software (Leica Microsystem) or ZEN Software (Zen 2 blue edition).

For F-actin visualizing, cells were fixed in 4% paraformaldehyde (Servicebio) after treatment. Nuclei were stained with DAPI (Beyotime), and the actin cytoskeleton was stained with Alexa Fluor 488 phalloidin (Servicebio, China; G1028). To visualize mitochondria, cells were loaded with 50 nm Mito-Tracker Red CMXRos (Beyotime Biotech, C1035) and Hoechst 33,342 (Beyotime Biotech, C1027) at 37 °C for 30 min to stain mitochondria and cell nuclear. The mean mitochondrial length was determined by measuring at least 10–20 individual mitochondria from cells obtained by fluorescence microscopy using Leica LAS AF software. The colocalization analyses of mitochondria and autophagosomes provide an indication of the initiation of mitophagy [26, 27]. To quantify early mitophagy, cells were transiently transfected with GFP-LC3 (Beyotime Biotech) and stained with 50 nM MitoTracker Red CMXRos after transfection at least 48 h [28, 29]. The percentage of LC3 puncta that colocalize with mitochondria was observed under Leica TCS SP8 confocal microscopy. Random fields of view were selected and imaged for further analysis. At least 20–50 cells in each of the three independent experiments were subjected to quantify the colocalization of LC3 puncta -MitoTracker in Image J software.

### Autophagy analysis

Autophagy was assessed through western blot analysis of autophagy, pCMV-GFP-LC3B plasmids (Beyotime Biotech, China) and transmission electron microscopy (TEM). Cells were transfected with GFP-LC3B plasmids for at least 48 h to visualize LC3 puncta. Green fluorescent protein (GFP) fluorescence was detected with the Zeiss LSM710 confocal microscope (20× lens) after transfection. For quantification, at least 20–50 GFP-positive cells in each group were used to calculated the number of LC3 puncta in each cell. Taking into account basal autophagy responses, those cells with more than 5 puncta were defined as autophagy active. Western blot analysis of autophagy and TEM procedures were described in detail in the following protocol.

### Western blot analysis

Western blot analysis was performed as previously described [22]. Cell lysates were subjected by SDS polyacrylamide gel electrophoresis (SDS-PAGE), transferred to polyvinylidene difluoride (PVDF) membranes (Immobilon P, Merck Millipore) and followed by immunoblot with corresponding antibodies. The following primary antibodies against Drp1 (Cell signal Technology, 5391 T, 1:1000), Mfn2 (Abcam, ab124773, 1:5000), LC3B (Cell signal Technology, 2775 S, 1:1000), SQSTM1 (Cell signal Technology, 5114 S, 1:1000) and β-actin (Abgent, AM1829b, 1:2000) were used. Horseradish peroxidase-coupled secondary antibodies were used for band detection with an ECL Plus Western blotting detection system (Tanon, China). The intensities of bands were quantified using ImageJ software. In addition, original data of western blot is reported as Original Data File.

### Transmission electron microscopy (TEM)

According to manufacturer’s instructions, samples were prepared and submitted to Wuhan Servicebio technology cooperation for transmission electron microscopy. Briefly, the cell samples were fixed in 1.25% glutaraldehyde/0.1 M phosphate buffer and postfixed in 1% OsO4/0.1 M phosphate buffer. Ultrathin sections of 50 nm were cut on a microtome, placed onto copper grids, stained with uranyl acetate and lead citrate, and examined under an electron microscope. Five random visual fields of each sample were analyzed.

### Seahorse assays

Extracellular acidification rate (ECAR) and oxygen consumption rate (OCR) were measured using the Seahorse XF96 Flux Analyzer (Seahorse Bioscience, USA). Data were analyzed by the Seahorse Wave Desktop Software (version 2.6, Seahorse Bioscience). The Seahorse assay was performed in accordance with manufacturer’s instructions (Seahorse Bioscience, USA). Briefly, cells were seeded at an appropriate density on Seahorse plate (6000, 10,000, 12,000 cells/well for 4T1-Luci, MDA-MB-231, MCF-7 cells, respectively), and incubated with ADT-OH for 24 h after the cells were adherent. For XF Cell Mito Stress Test kit (Agilent Technologies, 103015-100), OCR and ECAR were detected after injection of oligomycin (1.5 μM), Carbonyl cyanide-p-trifluoromethoxyphenylhydrazone (FCCP, 0.5 μM or 1 μM, respectively for MCF-7 and MB-231, 4t1-luci), and the combination of rotenone & antimycin (Rot/AA, 0.5 μM). For XF Glycolytic Rate Assay kit (Agilent Technologies, 103344-100), OCR and ECAR were detected after injection of the combination of rotenone & antimycin (Rot/AA, 0.5 μM), and 2-Deoxy-D-glucose (2-DG, 50 mmol/L). For XF Real-Time ATP Rate kit (Agilent Technologies, 103592-100), OCR and ECAR were detected after injection of oligomycin (1.5 μM), and the combination of rotenone & antimycin (Rot/AA, 0.5 μM). After the experiment, OCR and ECAR values were normalized to cell number or the protein amount in each well measured by Micro BCA Protein Assay kit (ThermoFisher Scientific, USA; 23235). Data were analyzed by the Seahorse Wave Desktop Software (version 2.6, Seahorse Bioscience).

### Mice

BALB/c, BALB/c nude mice (female, 6–8 weeks of age) were purchased from the Laboratory Animal Center of the Shanghai Institute of Planned Parenthood Research (Shanghai, China) and housed under specific pathogen-free (SPF) conditions for 1 week before the experiments. All experiments procedures have been approved by the Nanjing University Animal Care and Use Committee and performed in accordance with the guidelines of the Animal Protection Committee of Nanjing University (Nanjing, China). The animals were randomly allocated into experimental groups and the sample size used for animal experiments was 5 mice per group.

### Animal model and treatment

The effects of ADT-OH on tumorigenic and metastatic properties of two breast carcinoma cell lines, MDA-MB-231 and 4T1-Luci, were tested using an orthotopic model of tumor growth and spontaneous metastasis. For MDA-MB-231 orthotopic xenograft mouse model, 2 × 10^6^ cells were mixed with Matrigel (Corning, USA; 356234) at 1:1 ratio by volume and injected into the fourth mammary fat pads of anesthetized athymic Nu/Nu female mice, as described previously [30]. As shown in Supplementary Fig. S7A, treatment began when primary tumors were well-established (25–40 mm^3^ tumor volume). Tumor size was measured every second or third day with a digital vernier caliper and tumor volume was calculated using the following formula: tumor volume = (Width^2^ × Length)/2. Meanwhile, the body weight of the mice was measured and strictly monitored. Two or three weeks after starting the treatment, mice were euthanized, and organs were dissected, collected, weighed, photographed, and fixed in 10% formalin. Formalin-fixed paraffin-embedded tissue sections (5 μm thick) were stained with hematoxylin and eosin (H&E).

For 4T1-Luci syngeneic mouse model, 5 × 10^5^ cells used for orthotopic injection were injected into the fourth mammary fat pads of BALB/c female mice and the primary tumors were surgically resected on day 20 after inoculation to observe spontaneous lung metastasis [31, 32]. Using the IVIS- Spectrum in vivo imaging system (Caliper Life Sciences, USA), disease progression and therapeutic response were assessed through injection of 150 mg/kg of D-Luciferin (meilunbio, China). Ten minutes prior to imaging, mice were intraperitoneally injected with D-luciferin and were anaesthetized with an isoflurane/oxygen gas mixture five minutes later.

Additionally, experimental lung metastases were generated by intravenous (tail vein) injections of 1.2 × 10^5^ cells 4T1-Luci cells to assess the role of ADT-OH in tumor cell metastasis as previously described [33–35]. Disease progression in live animals was monitored via live animal imaging system as above-described. The timing of the imaging session and administration schedules were outlined in Fig. 8A.

### Statistical analysis

Statistical analysis was carried out using GraphPad Prism 8.0 (Graph Pad Software, USA). Data were expressed as means ± standard deviations (SD) or standard error mean (SEM) from at least three independent experiments. Student’s *t* test and one-way ANOVA were utilized for statistical analysis. Log-rank (Mantel-Cox) test was used for the survival curves analyses. Values of *p* < 0.05 were considered as statistically significant.

## Results

### ADT-OH significantly inhibits metastasis of TNBC cells in the absence of proliferation inhibition

**Fig. 1:**
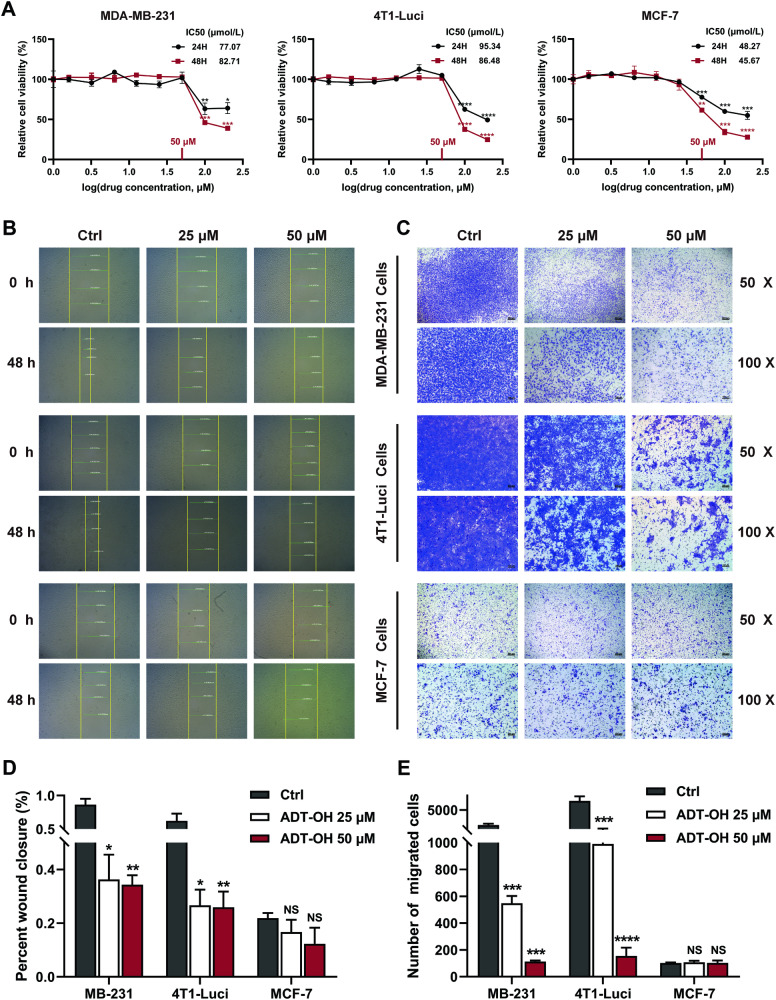
Effects of ADT-OH on the proliferation and metastatic ability of breast cancer cells. **A** Cell viability and IC50-values were determined by CCK-8 assay. MDA-MB-231, 4T1-Luci and MCF-7 breast cancer cells were incubated with ADT-OH (0–200 μM), and detected at 24, 48 h after treatment. **B** Representative micrographs of wound healing assays of MDA-MB-231, 4T1-Luci and MCF-7 cells at 0, 48 h in the presence of ADT-OH ( ≤ 50 μM) after scratching. original magnification, ×40. **C** Transwell assay was performed to further assess the migration ability of cells mentioned above using chambers uncoated with Matrigel. original magnification, ×50 (scale bar: 200 μm). original magnification, ×100 (scale bar: 100 μm). **D** The area of the wound was quantified (*n* = 3) by the ImageJ software and the percent of wound closure was measured. **E** The number of migrated cells per field (*n* = 5) of view was calculated by ImageJ software. Data are presented as mean ± SD of three independent experiments. NS not significant, *P < 0.05, **P < 0.01, ***P < 0.005, ****P < 0.001 compared with the control group.

In vitro efficacy of ADT-OH in breast cancer cells was analyzed in three breast cancer cell lines with different metastatic potential, including two typical TNBC cell lines, MDA-MB-231, 4T1-luci and one non-TNBC cell line, MCF-7. First, we performed CCK-8 assay to assess the cell viability and determine the suitable drug concentration. As shown in Fig. 1A, high concentrations of ADT-OH could significantly reduce the proliferation of three breast cancer cell lines. While ADT-OH inhibited the proliferation of all breast cancer cell lines in a dose-dependent manner, the IC50 (half-maximum inhibitory concentration) values were lower in MCF-7 cell line compared to the TNBC cell lines. These results suggest that MCF-7, the non-TNBC cell line, is more sensitive to ADT-OH than the TNBC cell lines. However, wound healing and transwell assays showed that treatment with ADT-OH ( ≤ 50 μM) could markedly inhibit the migration of TNBC cell lines in contrast to MCF-7 cell line, either vertical migration or lateral migration (Fig. 1B–E). In addition, it can be seen from Fig. 1B–E that non-TNBC cell line MCF-7 exhibited at least 5-fold lower migratory abilities when compared with MDA-MB-231 cell line and 4T1-Luci cell line. Taken together, these results confirm that the significant decrease in cell migration of TNBC cells with higher migratory abilities after treatment is due to the effect of ADT-OH (at relatively low concentrations) on cell migratory behavior but not due to the decrease in cellular proliferation.

### ADT-OH suppresses the formation of filopodia and lamellipodia of TNBC cells in the absence of affecting cell apoptosis and cell cycle

**Fig. 2:**
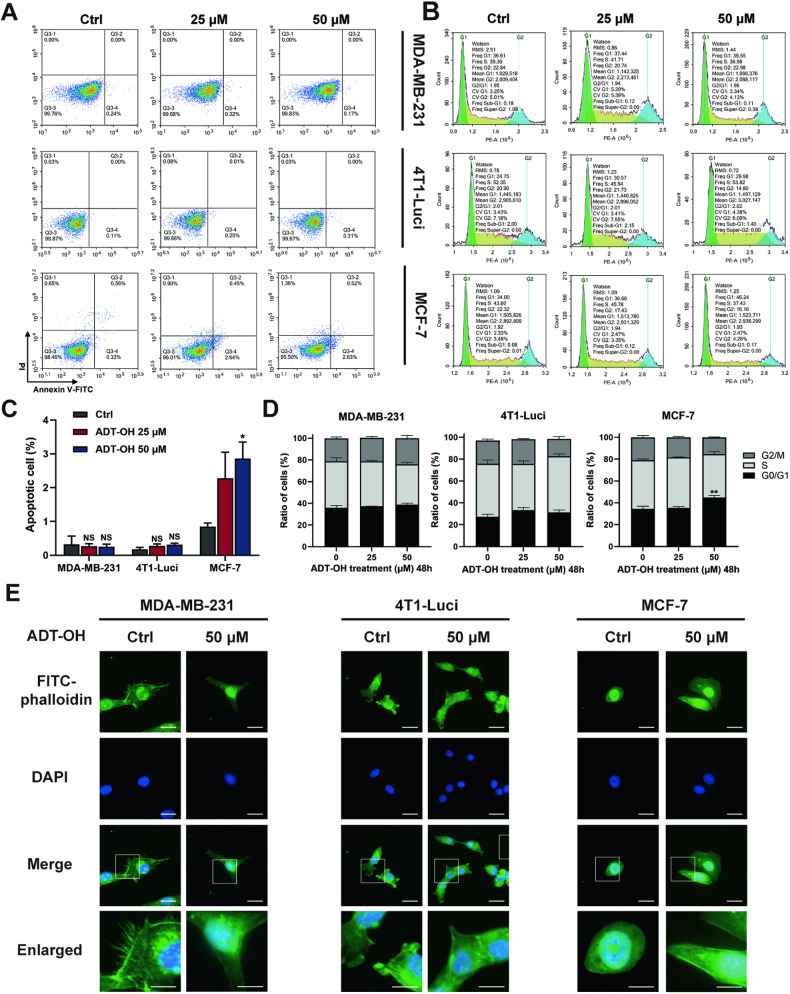
Effects of ADT-OH on the formation of pseudopodia and the cell apoptosis, cell cycle. **A** The cell apoptosis assay was determined with Annexin V/PI staining using flow cytometry analysis. MDA-MB-231, 4T1-Luci and MCF-7 breast cancer cells were treated with ADT-OH at different concentration (0–50 μM), and collected at 48 h after treatment for annexin V and propidium iodide double staining. **B** Cell cycle progression was determined with PI staining using flow cytometry analysis. **C**, **D** Quantitative analyses of apoptosis and cell cycle were determined as mean ± SD for triplicate experiments. Differences between treatment and control were statistically analyzed using Student’s *t* test (NS not significant, *P < 0.1 **P < 0.01, ***P < 0.005). **E** Cells were stained with Alexa Fluor 488 phalloidin for F-actin (green) and DAPI for Nuclei (blue), and then visualized with Zeiss confocal microscope. The quantitative analysis of cytoskeletal staining is presented in Supplementary Fig. S1. Scale bar, 25 μm; scale bar in enlarged image, 12.5 μm.

The effects of ADT-OH on the cell apoptosis and cell cycle of these cells were then determined by flow cytometric analysis. As shown in Fig. 2A–D, the presence of ADT-OH in MDA-MB-231 and 4T1-Luci cells caused no remarkable changes in cell apoptosis and the percent of cells in the G0/G1, S and G2/M phase, as compared with control cells. However, a relatively lower dose of ADT-OH (50 μM) could lead to apparent apoptosis and cell cycle change of MCF-7 cells. We next determined whether ADT-OH regulated the formation of filopodia and lamellipodia, the finger-like or flattened F-actin-rich membrane protrusions of migrating cells, which is a key structure for cancer cell migration and invasion both in vitro and in vivo [36, 37]. As shown in Fig. 2E and Supplementary Fig. S1, control cells, except MCF-7 cells, exhibited sheet-like or thread-like morphology throughout the cell edge, whereas ADT-OH-treated cells showed lesser branching and smoother edges. Collectively, these data demonstrate that ADT-OH inhibits invasion and migration of TNBC cells with minimal effects on cell proliferation, apoptosis and cell cycle at relatively low concentrations ( ≤ 50 μM).

Building upon these phenotypic data, we further wanted to address the possible molecular mechanism via which ADT-OH specifically inhibits TNBC cells migration without affecting cell proliferation, apoptosis and cell cycle. Thus, we need to further investigate to determine whether ADT-OH has effects on mitochondrial fission and autophagy flux and whether those effects result in the inhibition of migration ability in TNBC cells.

### ADT-OH induces mitochondrial elongation in TNBC cells

**Fig. 3:**
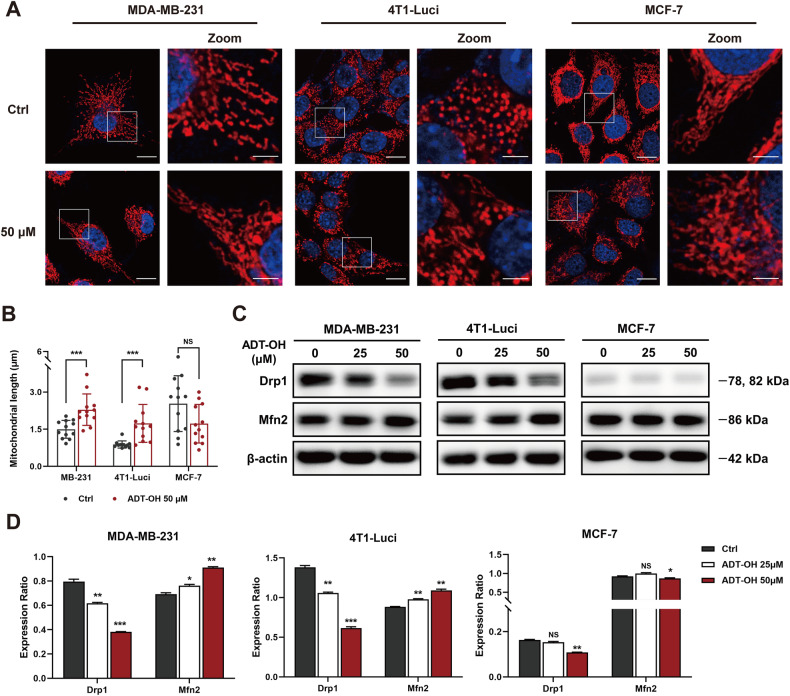
Effects of ADT-OH on mitochondrial dynamics in breast cancer cells. **A** Representative images of MDA-MB-231, 4T1-Luci and MCF-7 cells after 48 h treatment with ADT-OH, stained with MitoTracker Red, show mitochondrial morphology. scale bars, 15 μm; scale bar in enlarged image, 5 μm. **B** Quantitative analyses of mitochondrial length presented in panel (**A**) (*n* = 12). **C** Western blot analysis of Drp1 and Mfn2 expression levels in each group. β-Actin was used as an internal control. **D** Quantitative densitometry of relative expression level of Drp1 and Mfn2 in panel (**C**), normalized to the β-actin level. Data are presented as mean ± SD of three independent experiments. NS not significant, *P < 0.05, **P < 0.01, ***P < 0.005, ****P < 0.001 compared with the control group.

**Fig. 4:**
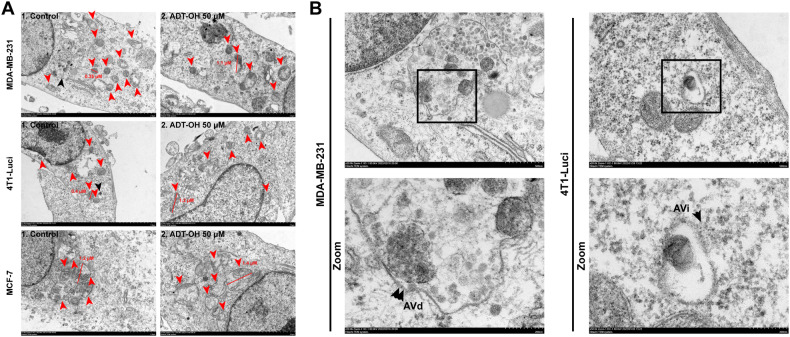
TEM analysis of mitochondrial morphology and ultrastructure after ADT-OH treatment. **A** Representative TEM images of control and 50 μM ADT-OH treated breast cancer cells. Quantitative analysis of length of mitochondria in plane (**A**) is presented in Supplementary Fig. S3. Red arrowheads in panel (**A**) indicate mitochondria, black arrowheads indicate autophagosome or autolysosome. **B** Magnification images of autophagosome or autolysosome indicating by black arrowheads in panel (**A**). In the enlarged view of the boxed area, single arrows denote autophagosomes (Avi), double arrows indicate autolysosomes (Avd).

Mitochondrial dynamics regulates migration and invasion of breast cancer cells and affects the formation of filopodia and lamellipodia without affecting the cell cycle and other cellular activities [18]. Therefore, mitochondria staining was first performed to detect the effects of ADT-OH on mitochondrial morphology and to elucidate the mechanism by which ADT-OH inhibits tumor cell migration. As shown in Fig. 3A, mitochondria in MCF-7 cells retained the tubular network-like structures whereas those in TNBC cells displayed a dot-shaped morphology. Mitochondrial length in TNBC cells were much shorter than MCF-7 cells, but this phenomenon was reversed after ADT-OH treatment, since the mitochondria in TNBC cells were elongated while the mitochondria in MCF-7 seem to be interrupted (Fig. 3A, B). Western blot assays showed that dynamin-related protein 1 (Drp1) was drastically decreased by about 50% in MDA-MB-231 and 4T1-Luci cells at the dose of 50 μM, as compared with control groups, whereas mitochondrial fusion protein (Mfn2) was significantly increased. Thus, treatment of TNBC cells with 50 μM ADT-OH resulted in interruption of mitochondrial fission and enhancement of mitochondrial fusion, which was associated with decreased expression of Drp1 and increased expression of Mfn2 (Fig. 3C, D). To further determine the changes of mitochondrial morphology in breast cancer cells treated with ADT-OH, transmission electron microscopy (TEM) was used to carry out an ultrastructural analysis in these cells. Interestingly, we observed some striking mitochondrial phenotypes in TEM pictures: a mitochondrion was undergoing division and a mitochondrion had just completed division, which showed typical mitochondrial fission processes and mitochondrial transport mediated by cytoskeleton after fission (Supplementary Fig. S2A, B). Most of the normal mitochondria in MDA-MB-231 and 4T1 cells range from 0.3–0.5 μM, just like the mitochondrion presented in Supplementary Fig. S2B. However, following 50 μM ADT-OH treatment, most dot-like mitochondria in MDA-MB-231 or 4T1-Luci cells were changed to elongated mitochondria with diameters over 1 µm (Fig. 4A, Supplementary Fig. S2C, D and Supplementary Fig. S3). Moreover, even the elongated mitochondria with exaggerated length also appeared, indicating that ADT-OH induces mitochondrial elongation caused by the interruption of mitochondrial fission, not mere association or aggregation of mitochondria (Supplementary Fig. S2C).

### ADT-OH decreases autophagy flux by suppressing autophagosome formation in TNBC cells

**Fig. 5:**
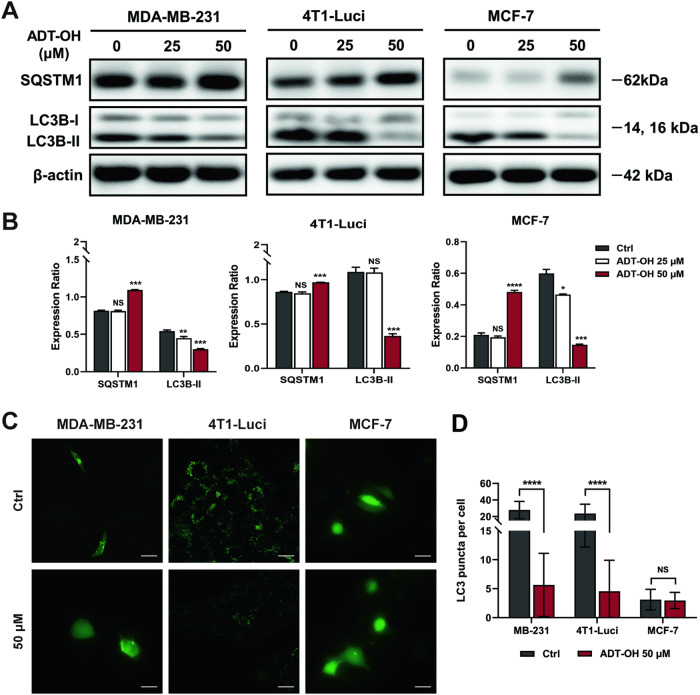
Effects of ADT-OH on autophagy in breast cancer cells. **A** Western blot analysis of LC3B-II and SQSTM1 expression levels in each group. β-Actin was used as an internal control. **B** Quantitative densitometry of relative expression level of LC3B-II and SQSTM1 in panel (**A**), normalized to the β-actin level. **C** MDA-MB-231, 4T1-Luci and MCF-7 cells expressing GFP-LC3B were treated with 50 μM ADT-OH for 48 h. Representative fluorescence images of GFP-LC3B puncta formation were observed using confocal microscopy. scale bar, 20 μm. **D** Quantification of GFP-LC3B puncta per cell treated as in panel (**C**). 20 to 50 cells from a pool of at least 10 images were analyzed per group. Data are presented as mean ± SD of three independent experiments. NS not significant, *P < 0.05, **P < 0.01, ***P < 0.005, ****P < 0.001 compared with the control group.

Compared with TNBC cells, Mfn2 is highly expressed in MCF-7 cells, whereas Drp1 is expressed at much lower levels, which means mitochondria were much more fragmented in TNBC cells than in MCF-7 cells (Fig. 3C, D). Overall, our findings are in line with the results reported in the literature [18]. As shown in Fig. 4A and Supplementary Fig. S3, the mitochondrial membrane was damaged and mitochondrial cristae decreased or disappeared in the three breast cancer cells after ADT-OH treatment, accompanied by the appearance of a small amount of mitochondrial vacuolization. Among them, MCF-7 cells were most obviously affected. Interestingly, the mitochondrial length determined by TEM pictures were not decreased in all groups mentioned above (Fig. 4A and Supplementary Fig. S3). Based on the TEM images in control groups, we observed that the number of autophagosomes and lysosomes in the tested TNBC cell lines were dramatically higher than in MCF-7 cell line (Fig. 4A). In line with published observations [8], we also confirmed that TNBC cancer cell lines, MDA-MB-231 and 4T1-Luci, displayed high basal levels of autophagy. In addition, we had identified the mitochondria in the autophagosomes or autolysosomes *via* the presence of mitochondrial double membrane and the remnant of mitochondrial cristae [38] (Fig. 4B). This observation indicated that TNBC cancer cell lines, MDA-MB-231 and 4T1-Luci, not only had active basal autophagy, but also the mitophagy (autophagy of mitochondria) at the same time. To determine whether ADT-OH affects autophagy in breast cancer cells, we compared the number of LC3 puncta in GFP-LC3 expressing cells before and after 50 μM ADT-OH administration. By analyzing the amount of LC3 puncta per cell with a confocal microscopy, we can quantify autophagosome and autophagy flux. In MDA-MB-231 and 4T1-Luci cells, treating cells with ADT-OH resulted in a significant decrease in GFP-LC3 puncta numbers, whereas there were no changes in MCF-7 cells (Fig. 5C, D). During autophagy, the soluble form of LC3B-I is modified and converted to a non-soluble form of LC3B-II, which is a hallmark of autophagy. Western blot analysis (Fig. 5A, B) showed that low dose of ADT-OH ( ≤ 50 μM) caused significant decreases in LC3B-II and pronounced accumulation of SQSTM1/p62 (reflecting lytic activity of autolysosome and autophagic flux [39]) in a dose-dependent manner in all breast cancer cell lines. Overall, we could determine that ADT-OH treatment decreased LC3B-II abundance and GFP-LC3B puncta formation and increased autophagic substrate SQSTM1/p62 levels in MDA-MB-231 and 4T1-Luci cells, indicating that the autophagy flux was decreased by suppressing autophagosome formation.

### ADT-OH downregulates mitophagy in TNBC cells

**Fig. 6:**
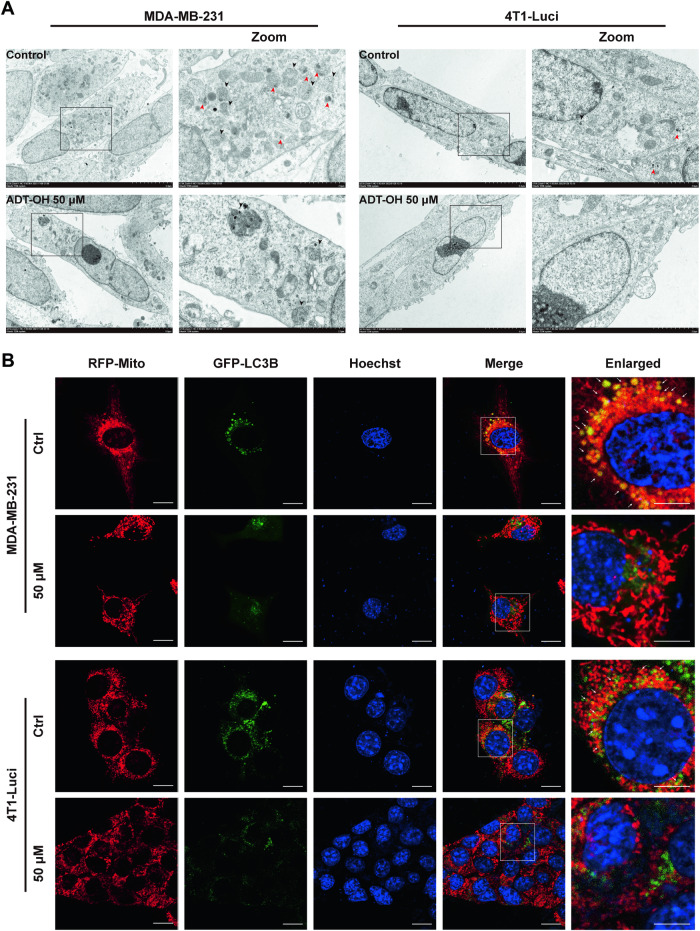
Effects of ADT-OH on mitophagy in MDA-MB-231 and 4T1 cells. **A** Representative TEM images of control and 50 μM ADT-OH treated breast cancer cells. Black arrowheads in panel (**A**) indicate autophagy, red arrowheads indicate mitophagy. **B** Representative colocalization images of mitochondria (MitoTracker Red) and autophagosomes (GFP-LC3B). Quantitative analysis of LC3 puncta - mitochondria colocalization per cell (colocalization area: LC3 puncta area) is presented in Supplementary Fig. S4B. For each picture, an enlargement of the merged image is displayed and the white arrowheads in the enlarged view of the white box highlight colocalization of mitochondria and autophagosome. Scale bars, 15 μm; scale bar in enlarged image, 7.5 μm.

To further determine the characteristics of autophagy of MDA-MB-231 and 4T1-Luci cells treated with ADT-OH, transmission electron microscopy (TEM) was used to perform ultrastructural analysis in these cells. As demonstrated in Fig. 6A and Supplementary Fig. S4A, the TEM studies revealed that there were much more autophagosomes and autolysosomes in control cells compared with 50 μM ADT-OH-treated TNBC cells, indicating the active autophagy flux was decreased. Furthermore, we could not observe the abnormal mitochondria or the remnant structure of mitochondria in limited autophagosomes or autolysosomes, indicating the 50 μM ADT-OH treatment not only decreased autophagosome formation and autophagy flux but also suppressed the mitophagy flux. Next, the colocalization of autophagosomes with mitochondria was observed to further confirm whether ADT-OH affected mitophagy. MDA-MB-231 and 4T1-Luci cells were transfected with the GFP-LC3B plasmaid and then stained with MitoTracker Red probe to label mitochondria and autophagosomes, respectively. Confocal microscope was used to analyze colocalization, we found the number of LC3 puncta colocalized with mitochondria per cell was significantly reduced compared with control cells (Fig. 6B and Supplementary Fig. S4B). To further confirm the effect of ADT-OH on autophagy, we assayed LC3-I/LC3B-II conversion in vitro using the late autophagy inhibitor Bafilomycin A1, which is recognized as the gold standard of autophagy detection [40, 41]. As shown in Supplementary Fig. S5, treatment of TNBC cells with the late autophagy inhibitor Bafilomycin A1 prevented intracellular lysosomal degradation. And when TNBC cells were exposed to both Bafilomycin A1 and ADT-OH, the accumulation of LC3B-II was significantly reduced. This indicated that ADT-OH significantly inhibited the activation of autophagic flux in MDA-MB-231 and 4T1 cells. These results further demonstrate that ADT-OH downregulates mitophagy in TNBC cells at the dose of 50 μM. Mitophagy was closely associated with mitochondrial dynamics comprising the regulation of mitochondrial morphology by mitochondrial fission and fusion [42]. Mitochondrial fission divides elongated mitochondria into pieces of appropriate size to be enveloped by autophagosomes, while mitochondrial fusion generates elongated mitochondria to prevent mitophagy [42, 43]. Since mitophagy is preceded by mitochondrial fission, the inhibition of mitochondrial fission may be responsible for the downregulation of mitophagy.

### ADT-OH inhibits mitochondrial function and enhanced glycolytic capacity of breast cancer cells

**Fig. 7:**
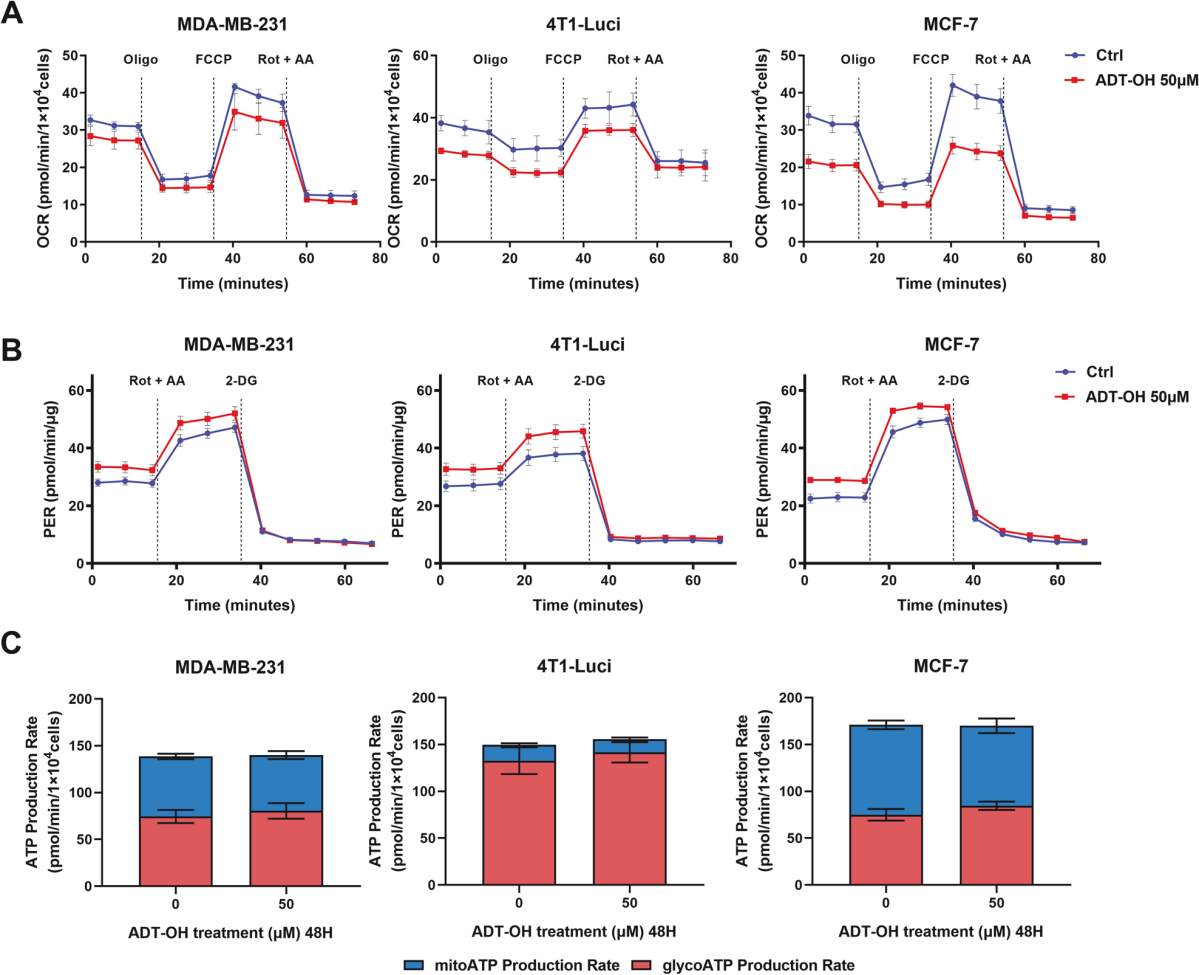
Effects of ADT-OH on oxidative phosphorylation and aerobic glycolysis in breast cancer cells. **A** Analysis of mitochondrial oxygen consumption rate (OCR) in control and ADT-OH treated (50 μM) breast cancer cells as determined by Cell Mito Stress Test. The oxygen consumption rate was normalized to total cell numbers. Quantitative analysis of area under the curve of OCR is presented in Supplementary Fig. S2B (*n* = 5). **B** Measurement of extracellular acidification rate (ECAR) in control and ADT-OH treated (50 μM) breast cancer cells as determined by Glycolytic Rate Assay. The extracellular acidification rate was normalized to total protein concentration. Quantitative analysis of area under the curve of ECAR is presented in Supplementary Fig. S2C (*n* = 5). **C** Mitochondrial and glycolytic ATP production rates in control and ADT-OH treated (50 μM) breast cancer cells as determined by XF Real-Time ATP Rate Assay.

Mitochondrial biogenesis, fission and fusion, and mitophagy play a critical role in regulating mitochondrial homeostasis balance and maintaining mitochondrial energetics [44]. In an attempt to assess energetic and metabolic changes in these breast cancer cells after ADT-OH treatment, we performed Cell Mito Stress Test, Glycolytic Rate Assay and XF Real-Time ATP Rate Assay. Based on our data, we observed that ADT-OH treatment in three breast cancer cell lines (MDA-MB-231, 4T1-Luci and MCF-7) significantly reduced basal respiration and spare respiratory capacity, suggesting that ADT-OH could inhibit mitochondrial oxidative function in those cells (Fig. 7A and Supplementary Fig. S6A). Surprisingly, ADT-OH treatment could inhibit mitochondrial oxidative function in 50 μM ADT-OH-treated TNBC cells, although we observed only the swollen or elongated mitochondria without obvious structural disruption. In addition, basial glycolysis, compensatory glycolysis and extracellular acidification were moderately increased, likely reflecting a compensatory response to the impaired mitochondrial function (Fig. 7B and Supplementary Fig. S6B). To further confirm the effect of ADT-OH on mitochondria ATP production and glycolytic ATP production, we performed Seahorse XF Real-Time ATP Rate assay. In agreement with our results above, ADT-OH reduced mitochondrial ATP production rate and enhanced glycolytic ATP production rate, while total ATP production rate was not affected compared to control cells. Furthermore, the relative contribution of glycolytic ATP production to the total ATP production was higher than mitochondria ATP production in all controls, especially in the TNBC controls, consistent with Warburg effect in cancer cells (Fig. 7C, D). In summary, we confirmed that ADT-OH inhibited mitochondrial function and enhanced glycolytic capacity in three breast cancer cells in a dose-dependent manner. In particular, the decreased mitochondrial function in 50 μM ADT-OH-treated TNBC cells attracted our attention.

Taken together, we concluded that highly fragmented mitochondria, active autophagy flux and mitophagy were essential to mitochondrial function, which supports the migration and invasion of TNBC cells. ADT-OH, through specific inhibition of both mitochondrial fission and autophagy/mitophagy flux, could significantly inhibit the migration and invasion of TNBC cells at relatively low concentrations ( ≤ 50 μM). According to the reported literature [18, 42, 44], we finally conclude that the inhibitions of mitochondrial fission and autophagy contribute to decrease in mitophagy flux, which eventually led to the decreased mitochondrial function and the inhibition of migration and invasion in TNBC cells. While the disruption of the mitochondrial structure observed in ADT-OH-treated MCF-7 cells and 100 μM ADT-OH-treated TNBC cells may be responsible for the inhibition of cell proliferation.

### ADT-OH significantly suppresses metastasis in TNBC orthotopic models and Tail vein metastasis model

**Fig. 8:**
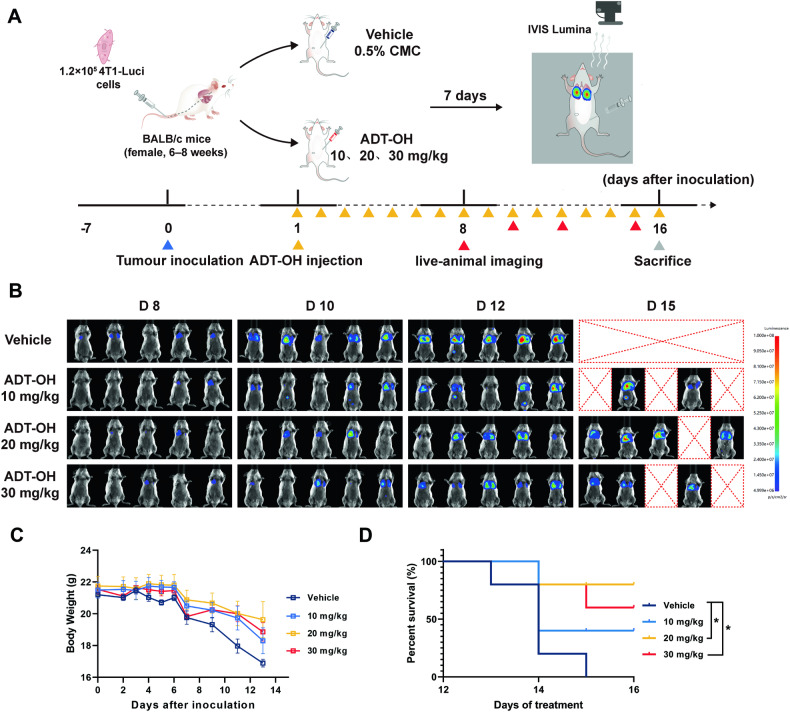
ADT-OH can significantly suppress the metastasis of 4T1-Luci cells in an experimental tail vein metastasis model. **A** Schematic diagram showing experiment design for in vivo drug evaluations. BALB/c mice (*n* = 5 per group) were injected on day 0 with 1.2 × 10^5^ 4T1-Luci cells in vivo and ADT-OH of different concentrations or vehicle control were administered on day 1 by intraperitoneal injection every day. On day 7 from the first treatment, mice were monitored for development of metastasis using in vivo bioluminescence imaging. **B** Bioluminescence images during the whole therapy. Quantitative analysis of bioluminescence in lung metastasis is presented in Supplementary Fig. S11A. **C** Body weights of mice were monitored over time. **D** Kaplan-Meier survival curve of mice treated with vehicle or ADT-OH. Log-rank (Mantel-Cox) test was used for the survival curves analyses. All data are presented as mean ± SEM. *P < 0.05 compared with the control group.

The above in vitro results indicate that ADT-OH offers a promising novel treatment strategy for targeting the metastasis in triple-negative breast cancer. We next investigated the therapeutic effect of ADT-OH on TNBC in the in vivo experiments. The TNBC cell lines, human MDA-MB-231breast cancer cells and mouse 4T1-Luci breast cancer cells, have been widely adopted to establish orthotopic xenograft mouse model and syngeneic mouse model [30–35]. Therefore, we next assessed the effects of ADT-OH on tumorigenic and metastatic properties of two breast carcinoma cell lines in vivo using two different TNBC models. Firstly, we investigated the anti-tumor ability of ADT-OH in MDA-MB-231 orthotopic xenograft mice. As shown in Supplementary Fig. S7A, 15 days post-tumor inoculation, mice received different doses of ADT-OH treatment. Using MDA-MB-231 orthotopic xenograft mouse model, we found ADT-OH had limited effect on the growth of primary tumors, but could significantly suppress the metastasis of MDA-MB-231 tumors (Supplementary Fig. S7C, D). There was a significant reduction in the metastatic liver area and number of liver surface metastatic nodules of mice with tumors in administration groups compared to tumors in control groups (Supplementary Fig. S7E, F). In addition, during the experiment, ADT-OH had no significant effect on body weight of mice at low, medium and high doses revealing a low drug toxicity (Supplementary Fig. S7B). We also administered excess ADT-OH (50 mg/kg) in normal mice for 50 days to observe the safety of ADT-OH. As shown in Supplementary Fig. S8, there was no significant difference in body weight and relative tissue weight (tissue/body weight) in normal mice with or without ADT-OH treatment. Therefore, the above results indicated that ADT-OH has fewer side effects or toxicity. To further assess the role of ADT-OH in tumor cell metastasis, we generated an experimental tail vein metastasis model and then monitored the development of tumor metastasis in vivo (Fig. 8A). As shown in Fig. 8B, tumor metastasized rapidly in mice of control group while the development of 4T1-Luci metastasis was considerably reduced in mice with ADT-OH treatment. Quantitative analysis of the tumor bioluminescence intensity further supported the anti-metastasis effects of ADT-OH (Fig. 8B and Supplementary Fig. S11A). The repeated ADT-OH administration every day over 2 weeks resulted in an increased survival time, while no mice survived in untreated groups (Fig. 8D). Long-term administration of ADT-OH not only did not cause significant adverse effects, but resulted in slower weight loss (Fig. 8C). Taken together, these data demonstrate that ADT-OH can significantly suppress the metastasis of 4T1-Luci cells to the lung and prolong survival compared with controls.

### CD-ADT-OH has better efficacy than ADT-OH alone in inhibiting tumor metastasis in vivo

A remarkable feature of the glycosylated nature of CDs is that they are not toxic to humans. Many CD-containing pharmaceutical compounds have been successfully approved by the FDA (Food and Drug Administration) and the EMA (European Medicines Agency) [45]. They can be widely used as excipients in drug structures and for improving the stability and bioavailability of poorly hydrophilic molecules. The fabrication of CD-ADT-OH host-guest complex was illustrated in Supplementary Fig. S9A. After co-incubation, ADT-OH was conjugated with β-cyclodextrins to eventually form CD-ADT-OH. Then CD-ADT-OH was characterized using Fourier Transform Infra-red (FT-IR) spectroscopy and UV/vis-absorption analysis. As shown in Supplementary Fig. S9B, there was no obvious difference of infrared chromatograph, but the ultraviolet absorption wavelength of ADT-OH was red-shifted after adsorbed with CD, indicating that the conjugation between CD and ADT-OH was simple intermolecular forces instead of forming chemical bonds. Next, wound healing assay showed that CD-ADT-OH significantly inhibited the migration of TNBC cell lines by reducing the scratch distance (Supplementary Fig. S9C, E). Additionally, the similar phenomenon was also observed in transwell assay (Supplementary Fig. S9D, F).

In order to better explore the anti-tumor effect of CD-ADT-OH, we haired the 4T1-Luci orthotopic model for validation. Currently, neoadjuvant chemotherapy and surgery are used most frequently to treat individuals with early TNBC [4]. To simulate this treatment regimen in mice, 4T1-Luci cells were implanted orthotopically and surgically removed 20 days after inoculation, when the tumor volume reached roughly 800 mm^3^ (Supplementary Fig. S10A). As shown in Supplementary Fig. S10B, ADT-OH treatment had limited effect on the growth of the primary tumor in 4T1 orthotopic model mice. We speculated that the limited effect of ADT-OH was most likely due to the poor solubility in an aqueous medium and lack of bioavailability. While, after complexation, CD-ADT-OH had increased the solubility and water stability (Supplementary Fig. S9A, B). Furthermore, CD-ADT-OH exhibited optimum inhibitory effect on tumor growth at the lowest concentration (Supplementary Fig. S10B, D and Supplementary Fig. S11B). Following tumor resection, the development of tumor metastasis and the duration of mice survival in control and CD-ADT-OH or ADT-OH-treated mice were monitored during the ensuing three weeks. Among mice undergoing protocol surgery, locoregional recurrence occurred in three of 31 mice, distributing in all groups except the CD-ADT-OH group. Excitingly, we found that ADT-OH treatment, especially CD-ADT-OH treatment, could significantly suppress not only the spontaneous metastasis but also the locoregional recurrence (Supplementary Figs. S10C, 11C). As shown in Supplementary Figs. S10C and 11C, the control group was detected the most severe bioluminescence signals of 4T1-Luci cells captured from orthotopic tumor and metastasis towards the end of the experiment. Moreover, CD-ADT-OH or ADT-OH treatment also increased the survival rate and prolonged the survival time of tumor-resected mice (Supplementary Fig. S10F). Notably, the results showed that 10 mg/kg CD-ADT-OH treatment exhibited the best curative effect and relatively little drug toxicity compared to 20 mg/kg and 30 mg/kg ADT-OH treatment group, although the latter groups with higher dose (Supplementary Fig. S10E, F). Therefore, CD-ADT-OH group made the satisfactory anti-tumor effect by suppressing mammary tumor metastasis. By using two different TNBC orthotopic models and one tail vein metastasis model, we fully demonstrated the inhibitory effects of ADT-OH, especially CD-ADT-OH, on cancer metastasis in vivo, offering a promising novel treatment strategy for TNBC metastasis.

## Discussion

TNBC is the most aggressive and the deadliest form of breast cancer, yet therapeutic strategies for TNBC are still lacking. Due to the multidrug resistance to conventional chemotherapies, the non-selective chemotherapy has limited efficacy for patients with relapsed/metastatic TNBC [5]. Compared with other breast cancer subtypes, the higher activities of autophagy and enhanced mitochondrial fission endow TNBC cells with the survival capacity and metastatic capacity in inhospitable microenvironment, including the stress environment generated by anticancer therapies [7, 8, 18]. Our in vitro experiments also proved the above conclusions. Moreover, we also demonstrated the presence of high level of mitophagy in TNBC cell lines, such as MDA-MB-231 and 4T1 cells. Instead of being generated from scratch, mitochondria proliferate via growth and fission of existing organelles and daughter mitochondria with different ΔΨ are generated after mitochondrial fission [15]. In those daughter mitochondria, healthy mitochondria with high ΔΨ will be transported to areas expected to have high ATP consumption or wait for subsequent cycle of fission and fusion, while dysfunctional mitochondria with low ΔΨ is likely to be removed by mitophagy [46]. However, more evidence from various studies supports that tumorigenesis depends on inhibition of mitophagy, tumor progression probably depends on functional mitophagy [47, 48], just like dual roles of autophagy in different stage of tumor development. Overall, compared with non-metastatic breast cancer cells, we found that metastatic breast cancer cells exhibit higher levels of autophagy, mitochondrial fission and mitophagy at the same time. All of those pathways promote plasticity of cancer cell metabolism for better adaption to the tumor microenvironment, which is also a major limitation of anticancer treatments [7, 49].

Interestingly, recent studies show that undifferentiated and rapidly proliferating stem cells have a metabolic profile reminiscent of aggressive cancer cells. Stem cells mainly rely primarily on aerobic glycolysis to generate energy and the mitochondria in these poorly differentiated cells are more fragmented and immature, containing less mtDNA and underdeveloped cristae structure [50]. Thus, utilization of OXPHOS is very low but glycolysis is increased [51]. From ATP production perspective, mitochondrial fission mediated by DRP1, which results in aerobic glycolysis, has no benefits for cells. But from the self-renewal perspective, mitochondrial fission is necessary for mtDNA selection and play a crucial role in developing and maintaining pluripotency. Sustained mitochondrial fragmentation exposed mutant genomes, and then mutant mitochondria are selected by mitophagy, which is the main pathway to eliminate dysfunctional mitochondria [52]. Similarly, compared with non-aggressive cancer cells, the aggressive cancer cells with more fragmented mitochondria exhibit poorer differentiation and higher self-renewal signaling [48]. It is becoming clear that mitochondrial dynamics plays an important role in controlling cell metabolism remodeling and mitophagy plays important roles in mitochondria quality control in a cell-autonomous manner [51]. Based on growing evidences that mitophagy pathways act as key regulators of dynamics and metabolic reprogramming, targeting mitophagy has been regarded as a promising therapeutic strategy for TNBC treatment [53]. However, a study reveals that mitophagy defects arising from loss of BNip3, mediating PRKN-independent mitophagy pathway [54], result in increased mammary tumor cell proliferation and metastasis. This is because in the absence of effective mitophagy and mitochondrial metabolism, BNip3 null tumor cells rely on macro-autophagy to survive by recycling macromolecules [55]. This sufficiently demonstrates the flexible metabolic mechanism supporting environment adaptation and survival of cancer cells. This also proves targeting only one pathway promoting plasticity of cancer cell metabolism is ineffective and combinatorial targeting is necessary. Mitophagy is a process of damaged mitochondria removal via autophagy [49]. Therefore, combined targeting of mitochondrial fission and autophagy may be the best way to target mitophagy and is necessary and sufficient to disrupt the plasticity of cancer cell metabolism.

In the present study, we found that ADT-OH, one of the most commonly used sustained-release H_2_S donors, inhibit autophagy and interrupts mitochondrial fission in breast cancer cells. MDA-MB-231 and 4T1 are highly metastatic TNBC cell lines with high autophagy and mitochondrial fission activity, whereas MCF-7 is non-metastatic breast cancer cell with low autophagy and mitochondrial fission activity. To better investigate the effects of ADT-OH on autophagy and mitochondrial fission activity in breast cancer cells, these cell lines were selected. Interestingly, the results showed that the effect of ADT-OH on TNBC cell phenotype and mechanism was variable at different concentrations, while the effect on MCF-7 cells was in a dose-dependent manner. Briefly, at the low concentrations, such as 50 μM, ADT-OH significantly inhibited metastasis of TNBC cells with little effect on cell proliferation, apoptosis, and cell cycle. While ADT-OH significantly inhibited cell proliferation without affecting cell metastasis in MCF-7 cells. The above results suggest that there might be two different mechanisms in TNBC cells and MCF-7 cell after ADT-OH treatment. Additionally, we found that ADT-OH suppress mitochondrial fission and autophagy flux, decreasing the mitophagy flux and mitochondrial function in TNBC cells. Moreover, TNBC cells treated with ADT-OH lost most plasticity of cancer cell metabolism and metastatic property, but obtained elevated glycolytic capacity. However, the increased glycolysis may not be simply compensation for reduced mitochondrial function. Evidence suggest that exposure to H_2_S significantly increased glycolysis, leading to increased lactate production and uncontrolled intracellular acidification [56]. Otherwise, we further demonstrated ADT-OH could significantly reduce metastatic capacity in vivo using three different TNBC models, MDA-MB-231 orthotopic xenograft model, 4T1-Luci orthotopic model and 4T1-Luci tail vein metastasis model. In conclusion, the in vitro and in vivo data validate the combinatorial targeting effect of mitochondrial fission and autophagy for the treatment of ADT-OH against TNBC metastasis.

Among a diversity of carrier systems, the efficiency of CD compounds as favorable delivery platforms for anti-tumor drug therapy has been well recognized. Numerous studies have shown that the binding of tumor-specific targeting ligands to drug-encapsulated CDs can improve the efficacy of therapeutic drugs/genes and reduce the side effects on healthy tissues [25, 45]. Here, we leveraged hydroxypropyl-β-cyclodextrin (CD) for systemic improving the solubility of ADT-OH. We demonstrate that engineered CD-ADT-OH exhibited effective tumor metastasis inhibition, while maintaining appreciable loading efficiency of the small molecule payload. Wound healing and transwell assays showed that CD-ADT-OH had potent effect on inhibiting TNBC cell migration without weakening the function of ADT-OH itself. Notably, CD-ADT-OH at a very low concentration of 10 mg/kg treatment showed the satisfactory anti-tumor effect on suppressing the TNBC metastasis of cells, which exhibited the best curative effect and relatively little drug toxicity compared to 20 mg/kg and 30 mg/kg ADT-OH treatment group. Systematic treatment of hydrogen sulfide donors or other small molecule compounds is not currently available yet, owing to dose-limiting adverse events which limit clinical efficacy and clinical application. Our study used cyclodextrin as a drug carrier for ADT-OH to improve its solubility and oncological efficacy, providing fresh perspectives on the clinical application of small molecule compounds and the creation of novel oncology medications.

## Conclusion

**Fig. 9:**
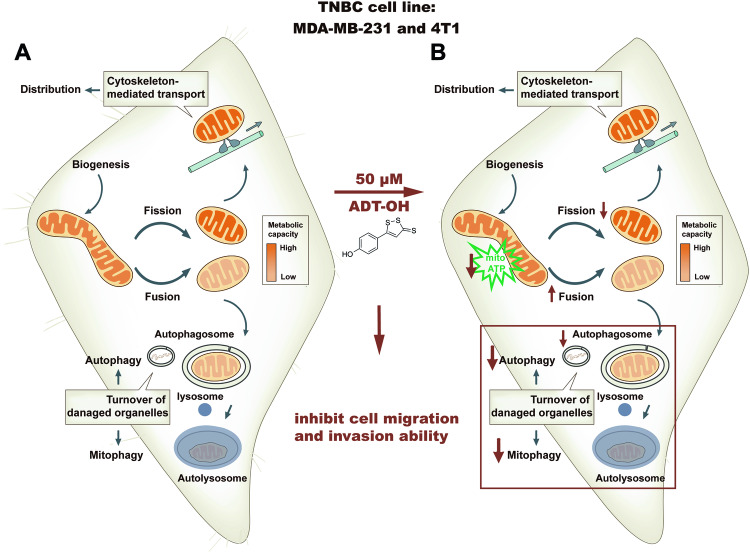
Graphical summary of proposed mechanism by which 50 μM ADT-OH significantly suppresses the metastasis of TNBC cells. **A** The initial state of mitochondrial dynamics and autophagy/mitophagy in MDA-MB-231 and 4T1 cells before ADT-OH administration. Mitochondria proliferate via growth and fission of existing organelles as opposed to de novo generate. After mitochondrial fission, daughter mitochondria with different ΔΨ are generated. In those daughter mitochondria, healthy mitochondria with high ΔΨ will be transported to areas, such as leading-edge of cancer cells, where are expected to have high ATP consumption, or wait for subsequent cycle of fission and fusion, while dysfunctional mitochondria with low ΔΨ are likely to be removed by mitophagy. **B** Alterations of intracellular mitochondrial dynamics and autophagy/mitophagy after ADT-OH administration. After 50 μM ADT-OH treatment, mitochondrial fission mediated by Drp1 and autophagy/mitophagy flux were significantly suppressed, which may be responsible for the inhibition of cell migration and invasion ability in MDA-MB-231 and 4T1 cells.

In summary, we here develop a novel strategy that enables the integration of targeting mitochondrial fission and autophagy pathways for the treatment of metastatic TNBC. By inhibiting mitochondrial fission and autophagy of TNBC cells, ADT-OH made an obvious inhibition effect on TNBC metastasis both in vivo and in vitro (Fig. 9). Otherwise, we endow CD-ADT-OH with pragmatic solubility for drug delivery and drug efficacy against TNBC metastasis. Considering the widespread upregulation of autophagy and mitochondrial fission in invasive tumor, the potential utility of ADT-OH extends beyond TNBC, and our findings indicated a promising prospect of CD-ADT-OH in exploration for clinical applications.

### Supplementary information

Supplementary Figures

Original Data File

## Data Availability

The data supporting the findings of this study are available from the corresponding author upon reasonable request.

## References

[1] Sung H, Ferlay J, Siegel RL, Laversanne M, Soerjomataram I, Jemal A (2021). Global Cancer Statistics 2020: GLOBOCAN estimates of incidence and mortality worldwide for 36 cancers in 185 countries. CA Cancer J Clin.

[2] Shah SP, Roth A, Goya R, Oloumi A, Ha G, Zhao Y (2012). The clonal and mutational evolution spectrum of primary triple-negative breast cancers. Nature.

[3] Liao C, Zhang Y, Fan C, Herring LE, Liu J, Locasale JW (2020). Identification of BBOX1 as a therapeutic target in triple-negative breast cancer. Cancer Discov.

[4] Won K-A, Spruck C (2020). Triple‑negative breast cancer therapy: current and future perspectives (Review). Int J Oncol.

[5] André F, Zielinski CC (2012). Optimal strategies for the treatment of metastatic triple-negative breast cancer with currently approved agents. Ann Oncol.

[6] Brown KK, Spinelli JB, Asara JM, Toker A (2017). Adaptive reprogramming of pyrimidine synthesis is a metabolic vulnerability in triple-negative breast cancer. Cancer Discov.

[7] Ferro F, Servais S, Besson P, Roger S, Dumas J-F, Brisson L (2020). Autophagy and mitophagy in cancer metabolic remodelling. Semin Cell Dev Biol.

[8] Lefort S, Joffre C, Kieffer Y, Givel A-M, Bourachot B, Zago G (2014). Inhibition of autophagy as a new means of improving chemotherapy efficiency in high-LC3B triple-negative breast cancers. Autophagy.

[9] Thomas S, Sharma N, Golden EB, Cho H, Agarwal P, Gaffney KJ (2012). Preferential killing of triple-negative breast cancer cells in vitro and in vivo when pharmacological aggravators of endoplasmic reticulum stress are combined with autophagy inhibitors. Cancer Lett.

[10] Hu J, Zhang Y, Jiang X, Zhang H, Gao Z, Li Y (2019). ROS-mediated activation and mitochondrial translocation of CaMKII contributes to Drp1-dependent mitochondrial fission and apoptosis in triple-negative breast cancer cells by isorhamnetin and chloroquine. J Exp Clin Cancer Res.

[11] Yeo SK, Paul R, Haas M, Wang C, Guan J-L (2018). Improved efficacy of mitochondrial disrupting agents upon inhibition of autophagy in a mouse model of BRCA1-deficient breast cancer. Autophagy.

[12] LeBleu VS, O’Connell JT, Gonzalez Herrera KN, Wikman H, Pantel K, Haigis MC (2014). PGC-1α mediates mitochondrial biogenesis and oxidative phosphorylation in cancer cells to promote metastasis. Nat Cell Biol.

[13] Chan DC (2020). Mitochondrial dynamics and its involvement in disease. Annu Rev Pathol.

[14] Youle RJ, van der Bliek AM (2012). Mitochondrial fission, fusion, and stress. Science.

[15] Westermann B (2010). Mitochondrial fusion and fission in cell life and death. Nat Rev Mol Cell Biol.

[16] Simula L, Nazio F, Campello S (2017). The mitochondrial dynamics in cancer and immune-surveillance. Semin Cancer Biol.

[17] Maycotte P, Marín-Hernández A, Goyri-Aguirre M, Anaya-Ruiz M, Reyes-Leyva J, Cortés-Hernández P (2017). Mitochondrial dynamics and cancer. Tumour Biol.

[18] Zhao J, Zhang J, Yu M, Xie Y, Huang Y, Wolff DW (2013). Mitochondrial dynamics regulates migration and invasion of breast cancer cells. Oncogene.

[19] Wu D, Wang H, Teng T, Duan S, Ji A, Li Y (2018). Hydrogen sulfide and autophagy: a double edged sword. Pharm Res.

[20] Guo W, Kan J-t, Cheng Z-y, Chen J-f, Shen Y-q, Xu J (2012). Hydrogen sulfide as an endogenous modulator in mitochondria and mitochondria dysfunction. Oxid Med Cell Longev.

[21] Jia J, Wang Z, Zhang M, Huang C, Song Y, Xu F (2020). SQR mediates therapeutic effects of HS by targeting mitochondrial electron transport to induce mitochondrial uncoupling. Sci Adv.

[22] Cai F, Xu H, Cao N, Zhang X, Liu J, Lu Y (2020). ADT-OH, a hydrogen sulfide-releasing donor, induces apoptosis and inhibits the development of melanoma in vivo by upregulating FADD. Cell Death Dis.

[23] Cai F-F, Xu H-R, Yu S-H, Li P, Lu Y-Y, Chen J (2021). ADT-OH inhibits malignant melanoma metastasis in mice via suppressing CSE/CBS and FAK/Paxillin signaling pathway. Acta Pharm Sin.

[24] Dong Q, Yang B, Han J-G, Zhang M-M, Liu W, Zhang X (2019). A novel hydrogen sulfide-releasing donor, HA-ADT, suppresses the growth of human breast cancer cells through inhibiting the PI3K/AKT/mTOR and Ras/Raf/MEK/ERK signaling pathways. Cancer Lett.

[25] Yi S, Liao R, Zhao W, Huang Y, He Y (2022). Multifunctional co-transport carriers based on cyclodextrin assembly for cancer synergistic therapy. Theranostics.

[26] Diot A, Hinks-Roberts A, Lodge T, Liao C, Dombi E, Morten K (2015). A novel quantitative assay of mitophagy: combining high content fluorescence microscopy and mitochondrial DNA load to quantify mitophagy and identify novel pharmacological tools against pathogenic heteroplasmic mtDNA. Pharm Res.

[27] Klionsky DJ, Abdel-Aziz AK, Abdelfatah S, Abdellatif M, Abdoli A, Abel S (2021). Guidelines for the use and interpretation of assays for monitoring autophagy (4th edition). Autophagy.

[28] Hsu P, Liu X, Zhang J, Wang H-G, Ye J-M, Shi Y (2015). Cardiolipin remodeling by TAZ/tafazzin is selectively required for the initiation of mitophagy. Autophagy.

[29] Vucicevic L, Misirkic-Marjanovic M, Paunovic V, Kravic-Stevovic T, Martinovic T, Ciric D (2014). Autophagy inhibition uncovers the neurotoxic action of the antipsychotic drug olanzapine. Autophagy.

[30] Kocatürk B, Versteeg HH. Orthotopic injection of breast cancer cells into the mammary fat pad of mice to study tumor growth. J Vis Exp. 2015. 10.3791/51967 PMC4354624 25742185

[31] Paschall AV, Liu K. An orthotopic mouse model of spontaneous breast cancer metastasis. J Vis Exp. 2016. 10.3791/54040 PMC5091834 27584043

[32] Zhang G-L, Zhang Y, Cao K-X, Wang X-M. Orthotopic injection of breast cancer cells into the mice mammary fat pad. J Vis Exp. 2019. 10.3791/58604 30735150

[33] Thies KA, Steck S, Knoblaugh SE, Sizemore ST. Pathological analysis of lung metastasis following lateral tail-vein injection of tumor cells. J Vis Exp. 2020. 10.3791/61270 32510518

[34] Warren JSA, Feustel PJ, Lamar JM. Combined use of tail vein metastasis assays and real-time in vivo imaging to quantify breast cancer metastatic colonization and burden in the lungs. J Vis Exp. 2019. 10.3791/60687 31904024

[35] Yue W, Chen L, Yu L, Zhou B, Yin H, Ren W (2019). Checkpoint blockade and nanosonosensitizer-augmented noninvasive sonodynamic therapy combination reduces tumour growth and metastases in mice. Nat Commun.

[36] Condeelis JS, Wyckoff JB, Bailly M, Pestell R, Lawrence D, Backer J (2001). Lamellipodia in invasion. Semin Cancer Biol.

[37] Jacquemet G, Hamidi H, Ivaska J (2015). Filopodia in cell adhesion, 3D migration and cancer cell invasion. Curr Opin Cell Biol.

[38] Chakraborty J, Caicci F, Roy M, Ziviani E (2020). Investigating mitochondrial autophagy by routine transmission electron microscopy: seeing is believing?. Pharm Res.

[39] Bjørkøy G, Lamark T, Brech A, Outzen H, Perander M, Overvatn A (2005). p62/SQSTM1 forms protein aggregates degraded by autophagy and has a protective effect on huntingtin-induced cell death. J Cell Biol.

[40] Kimmelman AC, White E (2017). Autophagy and tumor metabolism. Cell Metab.

[41] Zhang H, Gong Y, Wang Z, Jiang L, Chen R, Fan X (2014). Apelin inhibits the proliferation and migration of rat PASMCs via the activation of PI3K/Akt/mTOR signal and the inhibition of autophagy under hypoxia. J Cell Mol Med.

[42] Youle RJ, Narendra DP (2011). Mechanisms of mitophagy. Nat Rev Mol Cell Biol.

[43] Okamoto K, Kondo-Okamoto N, Ohsumi Y (2009). Mitochondria-anchored receptor Atg32 mediates degradation of mitochondria via selective autophagy. Dev Cell.

[44] Emery JM, Ortiz RM (2021). Mitofusin 2: a link between mitochondrial function and substrate metabolism?. Mitochondrion.

[45] Malhotra M, Gooding M, Evans JC, O’Driscoll D, Darcy R, O’Driscoll CM (2018). Cyclodextrin-siRNA conjugates as versatile gene silencing agents. Eur J Pharm Sci.

[46] Twig G, Elorza A, Molina AJA, Mohamed H, Wikstrom JD, Walzer G (2008). Fission and selective fusion govern mitochondrial segregation and elimination by autophagy. EMBO J.

[47] Chang JY, Yi H-S, Kim H-W, Shong M (2017). Dysregulation of mitophagy in carcinogenesis and tumor progression. Biochim Biophys Acta Bioenerg.

[48] Panigrahi DP, Praharaj PP, Bhol CS, Mahapatra KK, Patra S, Behera BP (2020). The emerging, multifaceted role of mitophagy in cancer and cancer therapeutics. Semin Cancer Biol.

[49] Vara-Perez M, Felipe-Abrio B, Agostinis P (2019). Mitophagy in cancer: a tale of adaptation. Cells.

[50] Zhang J, Khvorostov I, Hong JS, Oktay Y, Vergnes L, Nuebel E (2011). UCP2 regulates energy metabolism and differentiation potential of human pluripotent stem cells. EMBO J.

[51] Chen H, Chan DC (2017). Mitochondrial dynamics in regulating the unique phenotypes of cancer and stem cells. Cell Metab.

[52] Lieber T, Jeedigunta SP, Palozzi JM, Lehmann R, Hurd TR (2019). Mitochondrial fragmentation drives selective removal of deleterious mtDNA in the germline. Nature.

[53] Poole LP, Macleod KF (2021). Mitophagy in tumorigenesis and metastasis. Cell Mol Life Sci.

[54] Lin Q, Li S, Jiang N, Jin H, Shao X, Zhu X (2021). Inhibiting NLRP3 inflammasome attenuates apoptosis in contrast-induced acute kidney injury through the upregulation of HIF1A and BNIP3-mediated mitophagy. Autophagy.

[55] Chourasia AH, Tracy K, Frankenberger C, Boland ML, Sharifi MN, Drake LE (2015). Mitophagy defects arising from BNip3 loss promote mammary tumor progression to metastasis. EMBO Rep.

[56] Lee ZW, Teo XY, Tay EYW, Tan CH, Hagen T, Moore PK (2014). Utilizing hydrogen sulfide as a novel anti-cancer agent by targeting cancer glycolysis and pH imbalance. Br J Pharm.

