# Supplementary material for: ADT-OH exhibits anti-metastatic activity on triple-negative breast cancer by combinatorial targeting of autophagy and mitochondrial fission
Source: Cell Death Dis. 2024 Jun 28;15(6):463. doi: 10.1038/s41419-024-06829-w (PMC11213877; doi:10.1038/s41419-024-06829-w)
Supplement: Supplementary file 1 — Supplementary Figures [file 41419_2024_6829_MOESM1_ESM.pdf]

ADT-OH exhibits anti-metastatic activity on triple-negative breast cancer by combinatorial targeting of autophagy and mitochondrial fission

Shihui Yu[1,#], Zhiting Cao[3,#], Fangfang Cai[1,3,#], Yingying Yao[1], Xiaoyao Chang[1], Xiaoyang Wang[1], Hongqin Zhuang[1,*], Zi-Chun Hua[1,2,3,*]

[1]The State Key Laboratory of Pharmaceutical Biotechnology, College of Life Sciences, Nanjing University, Nanjing, P. R. China

[2]Changzhou High-Tech Research Institute of Nanjing University and Jiangsu TargetPharma Laboratories Inc., Changzhou 213164, P. R. China

[3]School of Biopharmacy, China Pharmaceutical University, Nanjing 211198, China

[#]These authors contributed equally to this work.

***Corresponding authors:**

Hongqin Zhuang, School of Life Sciences, Nanjing University, 163 Xianlin Blvd., Nanjing 210023, China. Phone: 86-25-89683692; Fax: 86-25-83324605, E-mail: hqzhuang@nju.edu.cn

Zi-Chun Hua, School of Life Sciences, Nanjing University, 163 Xianlin Blvd., Nanjing 210023, China. Phone: 86-25-89683692; Fax: 86-25-83324605, E-mail: hzc1117@nju.edu.cn

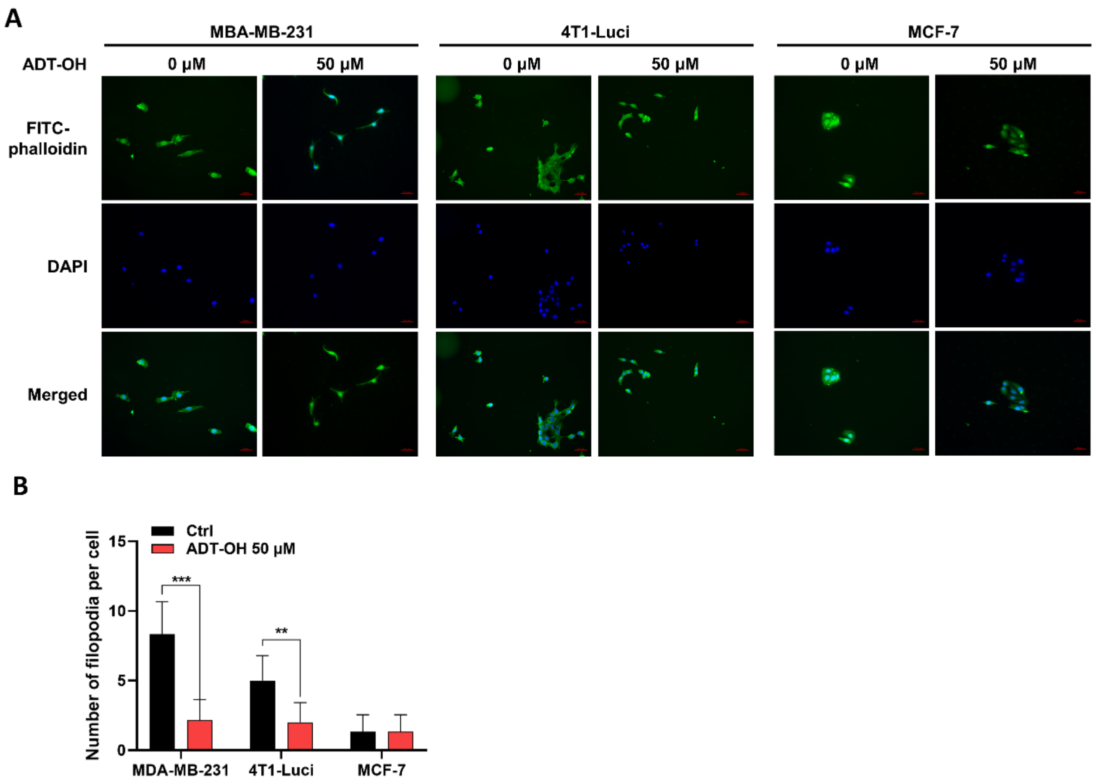

Supplementary Fig. 1 (A) Representative images of F-actin immunofluorescence in MDA-MB-231, 4T1 and MCF-7 cells after treated with or without 50 μM ADT-OH. Scale bars correspond to 50 μm. (**B**) The quantitative analysis of cytoskeletal staining after ADT-OH treatment presented in Fig. 2E.

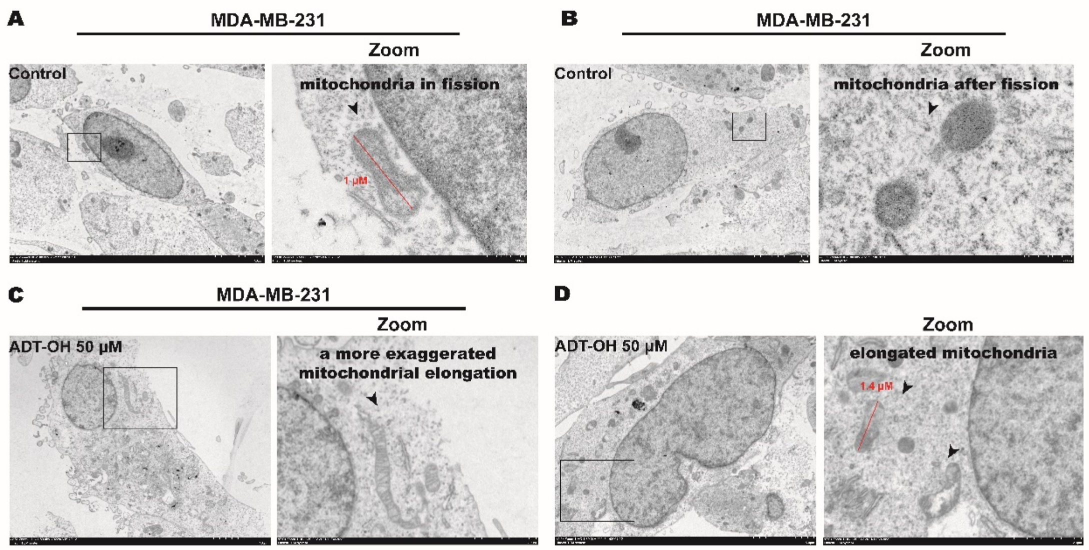

Supplementary Fig. 2 ADT-OH interrupts mitochondrial fission and inhibits mitophagy in TNBC cells.

Representative TEM images of control **(A-B)** and ADT-OH (50 μM) treated MDA-MB-231 cells **(C-D)**. For each image, an enlargement of the TEM image is displayed, showing the details of normal or abnormal mitochondria. **(A)** The black arrowhead in panel (a) shows a mitochondrion undergoing division. **(B)** The black arrowhead in panel (B) shows a mitochondrion have just completed division. **(C)** The black arrowhead in panel (C) shows a more exaggerated mitochondrial elongation. **(D)** The black arrowheads in panel (d) show elongated mitochondria.

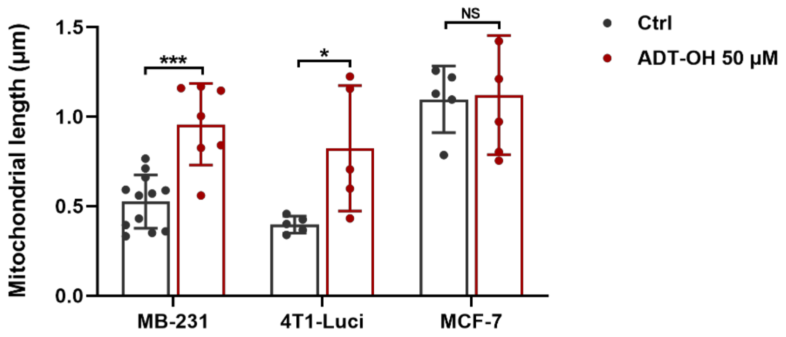

Supplementary Fig. 3 Quantitative analysis of the length of mitochondrial presented in Fig. 4A.

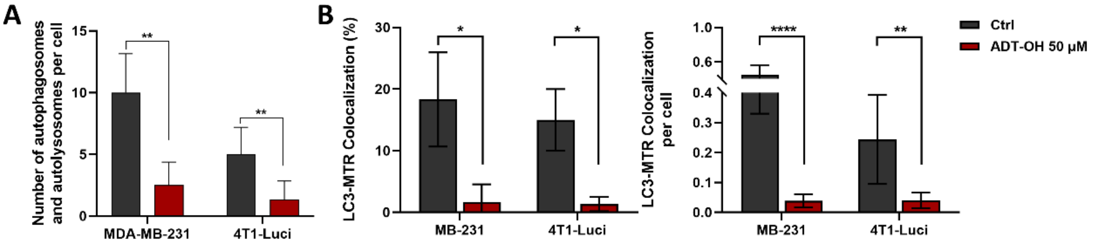

Supplementary Fig. 4 (A) Quantification of the number of autophagosomes and autolysosomes in MDA-MB-231 and 4T1-Lucci cells. **(B)** Quantification of colocalization of GFP-LC3/MitoTracker of the data presented in Fig. 6B. The left panel shows the percentage of cells with LC3 and mitochondria (MitoTracker Red) colocalization. In right panel, the quantitative analysis of colocalization of GFP-LC3 puncta with mitochondria per cell is expressed as Manders coefficients, which indicates colocalization area/LC3 puncta area per cell. 20 to 50 cells from a pool of at least 10 images were analyzed per group. Quantitative analysis was carried out using ImageJ software, and data are presented as mean ± SD. NS = not significant, *P < 0.05, **P < 0.01, ***P < 0.005, ****P < 0.001 compared with the control group.

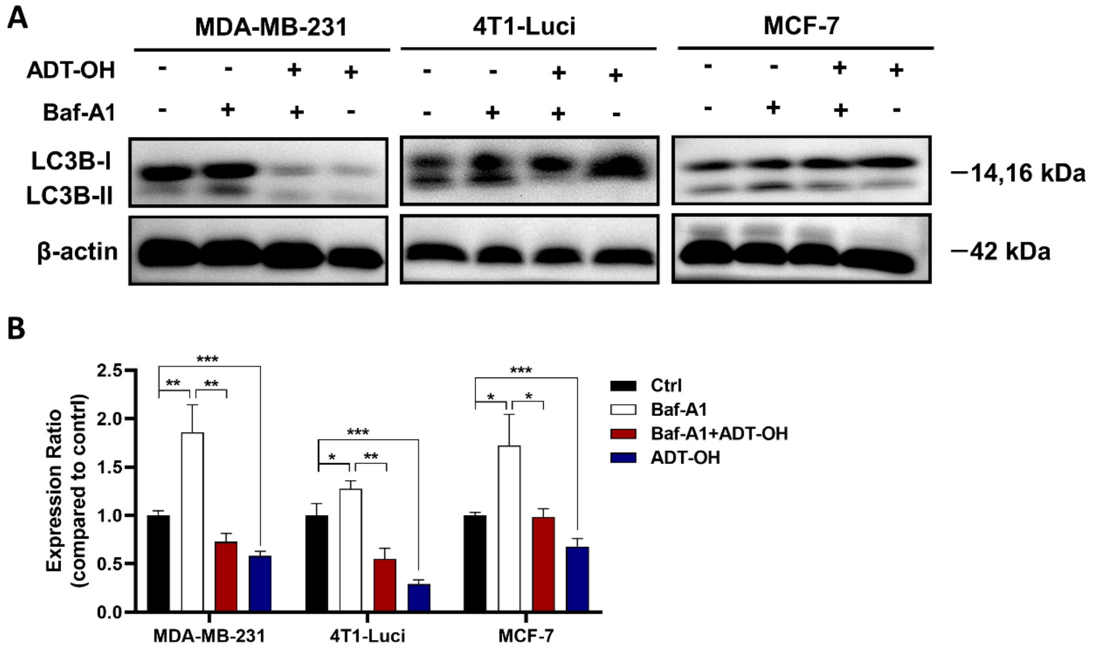

Supplementary Fig. 5 ADT-OH significantly inhibited the activation of autophagic flux in MDA-MB-231 and 4T1 cells. (A) Western Blot assay was used to analyze the autophagic flux in MDA-MB-231 and MCF-7 cells treated with ADT-OH (50 μM) or bafilomycin A1 (1 μM). **(B)** Quantitative densitometry of relative expression level of LC3B-II in panel (A), and data are presented as mean ± SD. n=3, *P < 0.05, **P < 0.01, ***P < 0.005 compared with the control group.

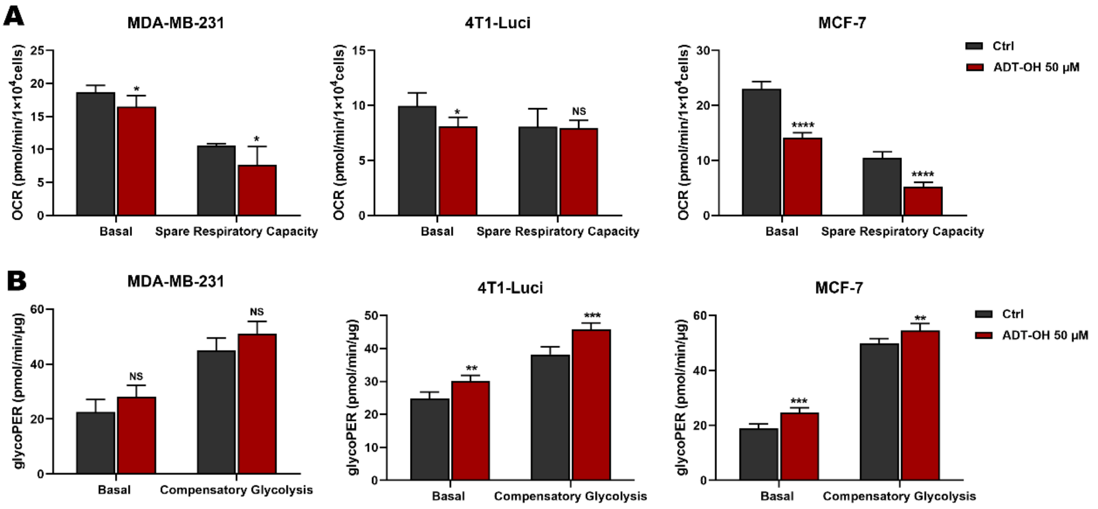

Supplementary Fig. 6 ADT-OH inhibits mitochondrial function and enhanced glycolytic capacity in three kinds of breast cancer cells in a dose-dependent manner. **(A)** Quantitative analysis of area under the curve of OCR presented in Fig. 7A. Statistical analysis was done for the basal respiration and spare respiratory capacity (n = 5). **(B)** Quantitative analysis of area under the curve of ECAR presented in Fig. 7B. Statistical analysis was done for the basial glycolysis and compensatory glycolysis (n = 5). Data are shown as mean ± SD. NS = not significant, *P < 0.05, **P < 0.01, ***P < 0.005, ****P < 0.001 compared with the control group.

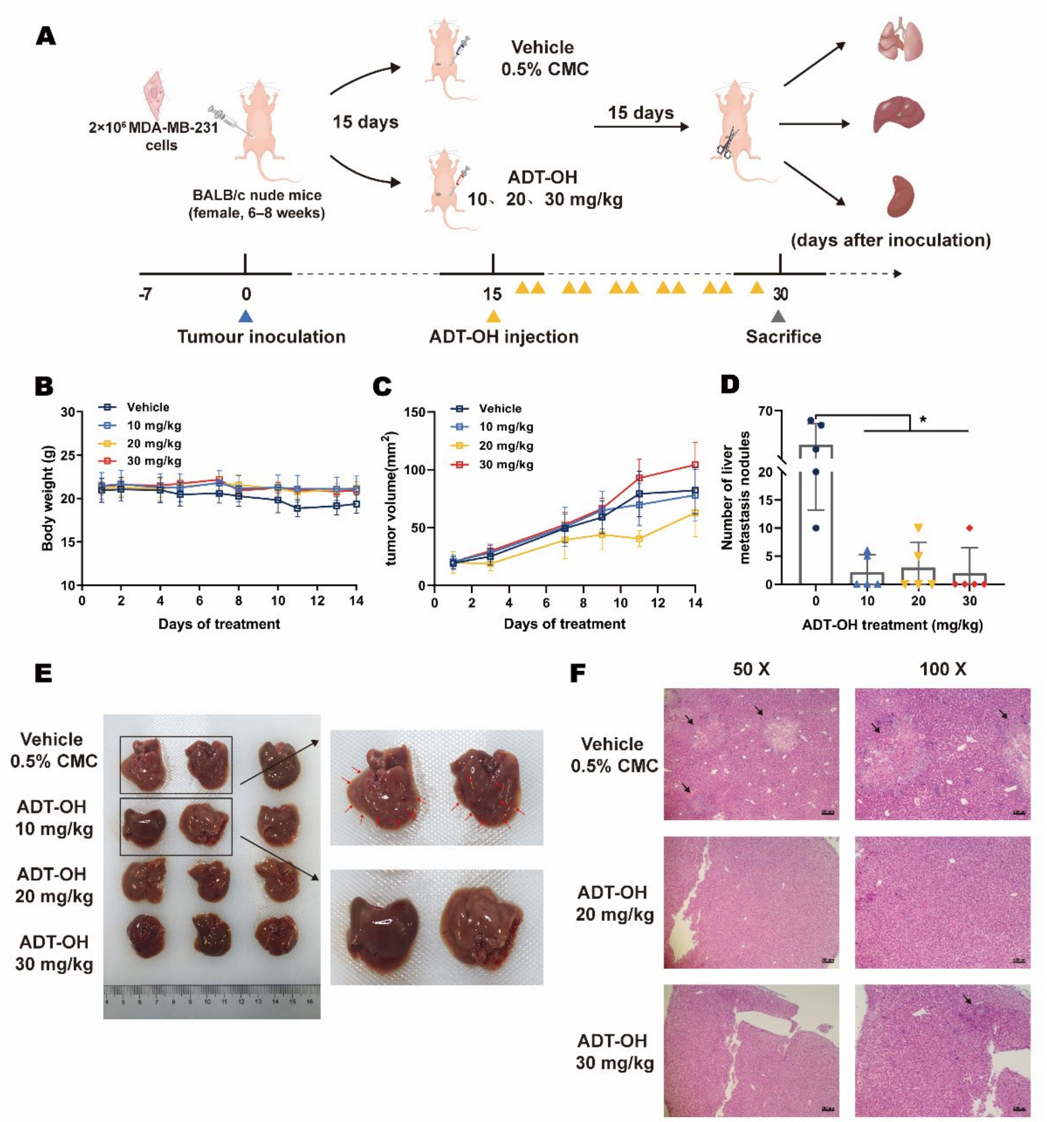

Supplementary Fig. 7 ADT-OH can significantly suppress the metastasis of MDA-MB-231 cells in an orthotopic xenograft mouse model.

(A) Schematic diagram showing experiment design for in vivo drug evaluations. BALB/c nude mice (n = 5 per group) were injected on day 0 with 2 × 10^6 MDA-MB-231 cells into the fourth mammary fat pads and ADT-OH of different concentrations or vehicle control were administered on day 15 by intraperitoneal injection. On day 15 from the first treatment, mice were euthanized for analysis. **(B)** Body weights of mice were monitored over time. **(C)** Tumor progression curves of different experimental groups over the experimental time course. Tumor volume was calculated using the following formula: tumor volume = (Width^2 × Length)/2. **(D-E)** Livers of mice were collected from animals euthanized and examined for metastases. Red arrowheads

indicate the metastatic nodules in the liver surface. Quantitative analysis of number of metastatic nodules per liver was presented in **(D)**. **(F)** Representative images of H&E-stained liver sections. Black arrowheads point to metastatic areas. Scale bar, 200 μm; scale bar in enlarged image, 100 μm. All data are presented as mean ± SEM. *$P < 0.05$ compared with the control group.

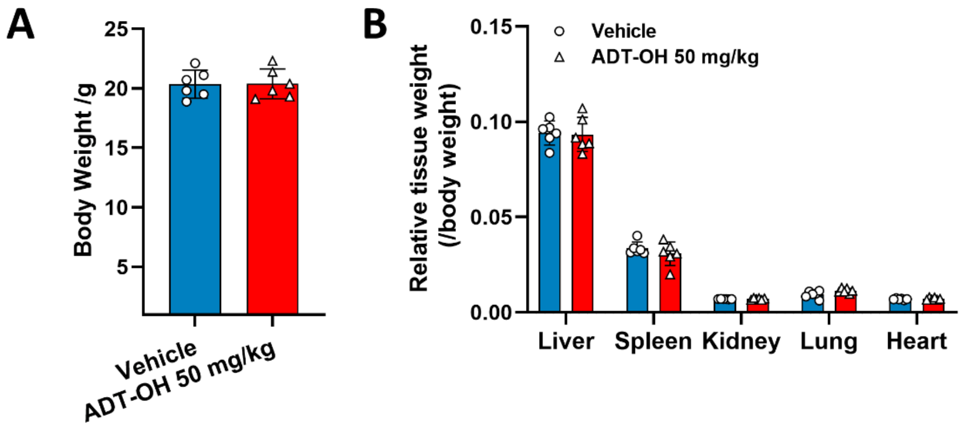

Supplementary Fig. 8 Normal mice were administered Vehicle (0.5% CMC) or ADT-OH (50 mg/kg) for 50 days. (**A**) The body weight of mice was monitored after euthanasia. (**B**) Relative weight of livers, spleens, kidneys, hearts and lungs of mice.

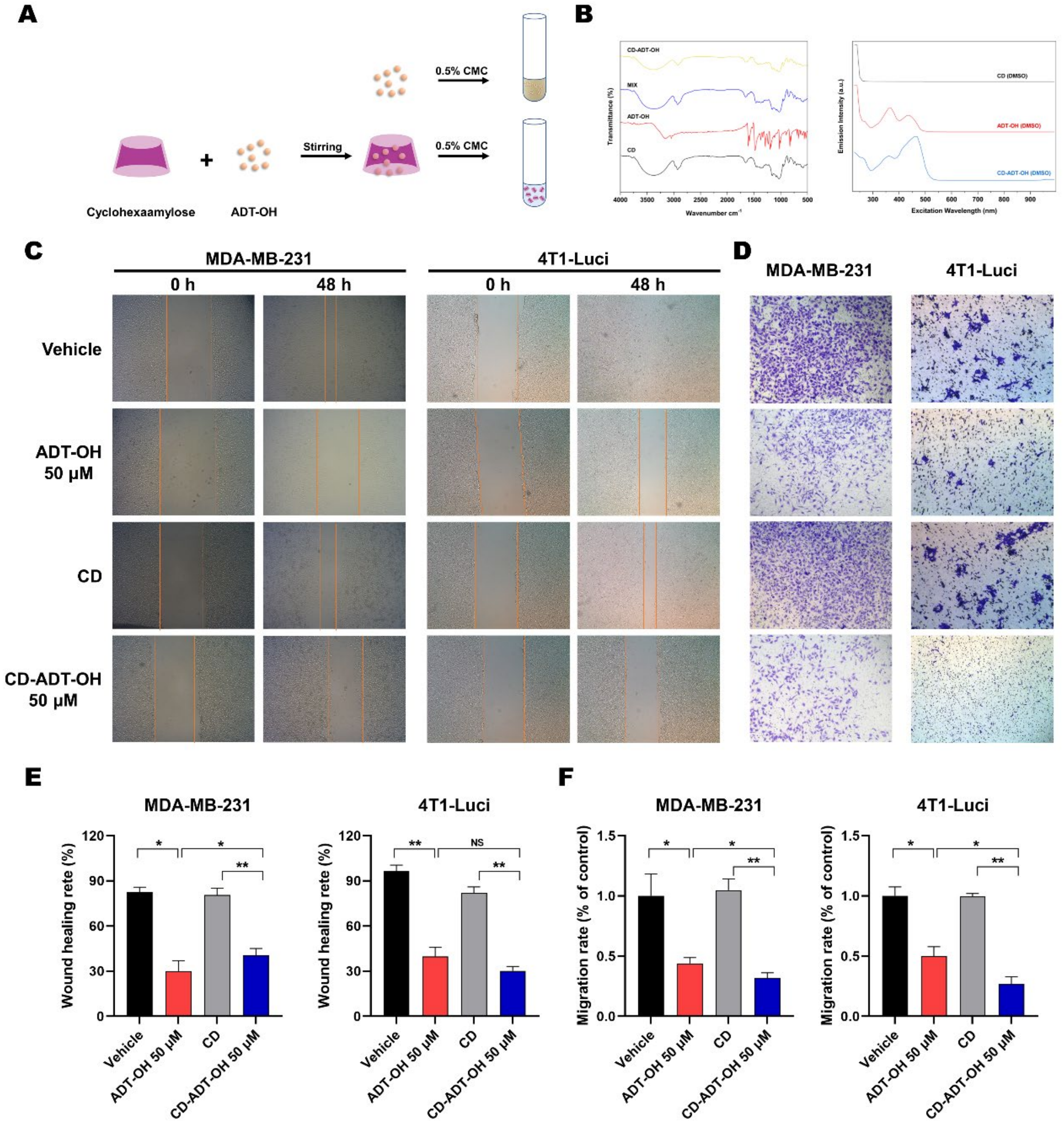

Supplementary Fig. 9 Effects of CD-ADT-OH on the metastatic ability of breast cancer cells.

(A) Cartoon of CD-ADT-OH or ADT-OH preparation process. **(B)** Fourier Transform Infra-red spectroscopy and UV/vis-absorption analysis of ADT-OH before and after absorption with CD. FT-IR spectra of CD, ADT-OH, the mixture of CD and ADT-OH and CD-ADT-OH. There was no obvious difference of infrared chromatograph since the conjugation between CD and ADT-OH was simple intermolecular forces instead of forming chemical bonds (left); UV-vis spectra of CD, ADT-OH and CD-ADT-OH (all dissolved in DMSO). The ultraviolet absorption wavelength of ADT-OH was red-

shifted after adsorbed with CD, which was caused by the conjugation effect between CD and ADT-OH (right). **(C)** Representative micrographs of wound healing assays of MDA-MB-231 and 4T1-Luci cells at 0, 48 h in the presence of CD-ADT-OH (50 μM) after scratching. original magnification, ×40. **(D)** Transwell assay was performed to further assess the migration ability of cells mentioned above using chambers. original magnification, ×50. original magnification. **(E)** The area of the wound was quantified (n=3) by the ImageJ software and the percent of wound closure was measured. **(F)** The number of migrated cells per field (n=5) of view was calculated by ImageJ software. Data are presented as mean ± SD of three independent experiments. NS = not significant, $^*P < 0.05$, $^{**}P < 0.01$, $^{***}P < 0.005$, $^{****}P < 0.001$ compared with the control group.

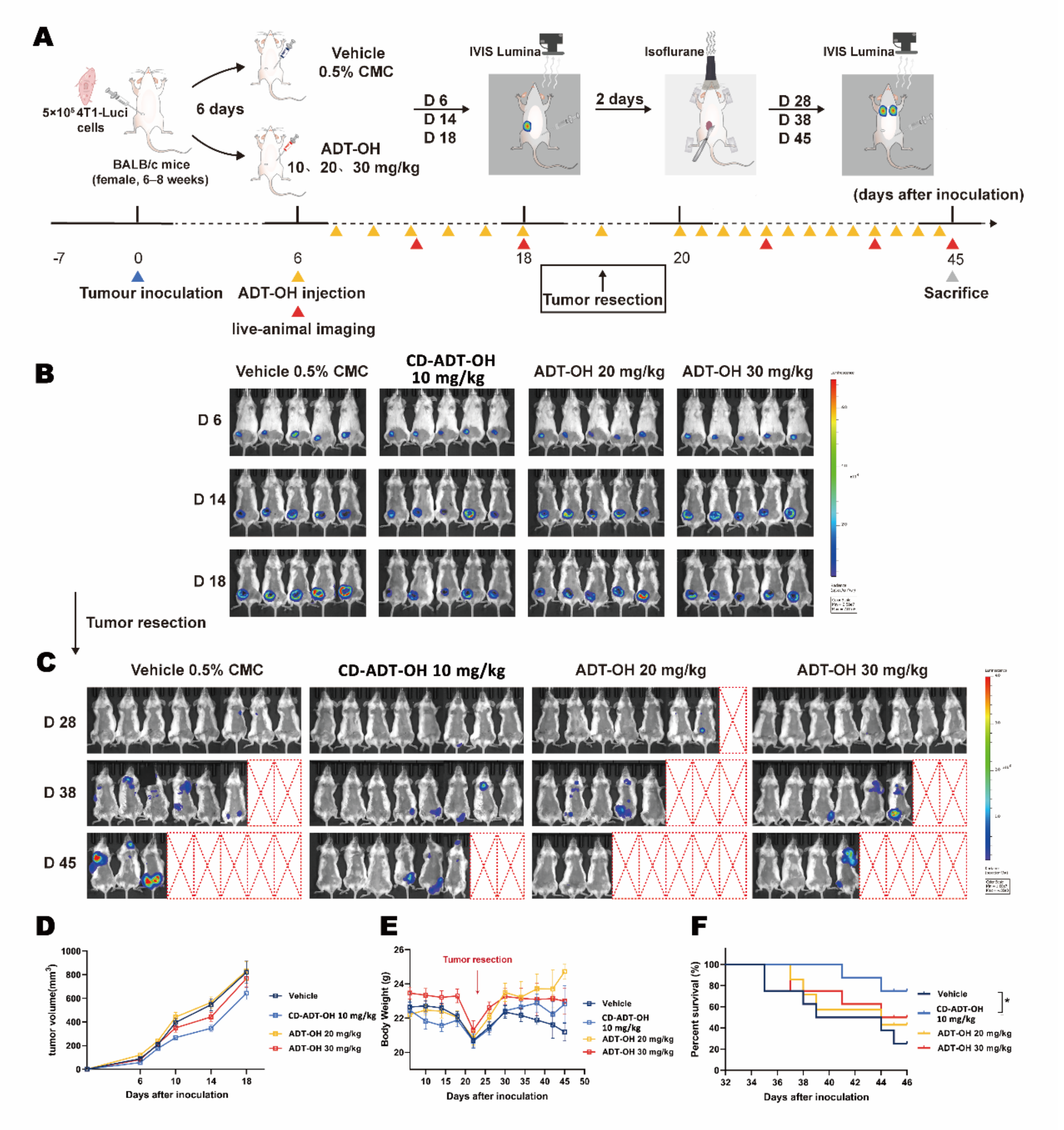

Supplementary Fig. 10 ADT-OH can significantly suppress the metastasis of 4T1-Luci cells in a syngeneic orthotopic model

(A) Schematic diagram showing experiment design for in vivo drug evaluations. BALB/c mice (n = 8 per group) were injected on day 0 with 5 × 10^5 4T1-Luci cells into the fourth mammary fat pads and ADT-OH of different concentrations or vehicle control were administered on day 6 by intraperitoneal injection. At the same time, mice were subjected to bioluminescence imaging for to detect primary tumor development. On day 20 from the tumor inoculation, 4T1-Luci tumors were surgically removed to monitor spontaneous metastasis using bioluminescence imaging when the tumor volume reached about 800 mm^3. **(B-C)** Bioluminescence images during the whole

therapy. **(D)** Tumor progression curves of different experimental groups over the experimental time course. Tumor volume was calculated using the following formula: tumor volume = (Width^2 × Length)/2. **(E)** Body weights of mice were monitored over time. **(F)** Kaplan-Meier survival curve of mice treated with vehicle or ADT-OH. Log-rank (Mantel-Cox) test was used for the survival curves analyses. All data are presented as mean ± SEM. *P < 0.05 compared with the control group.

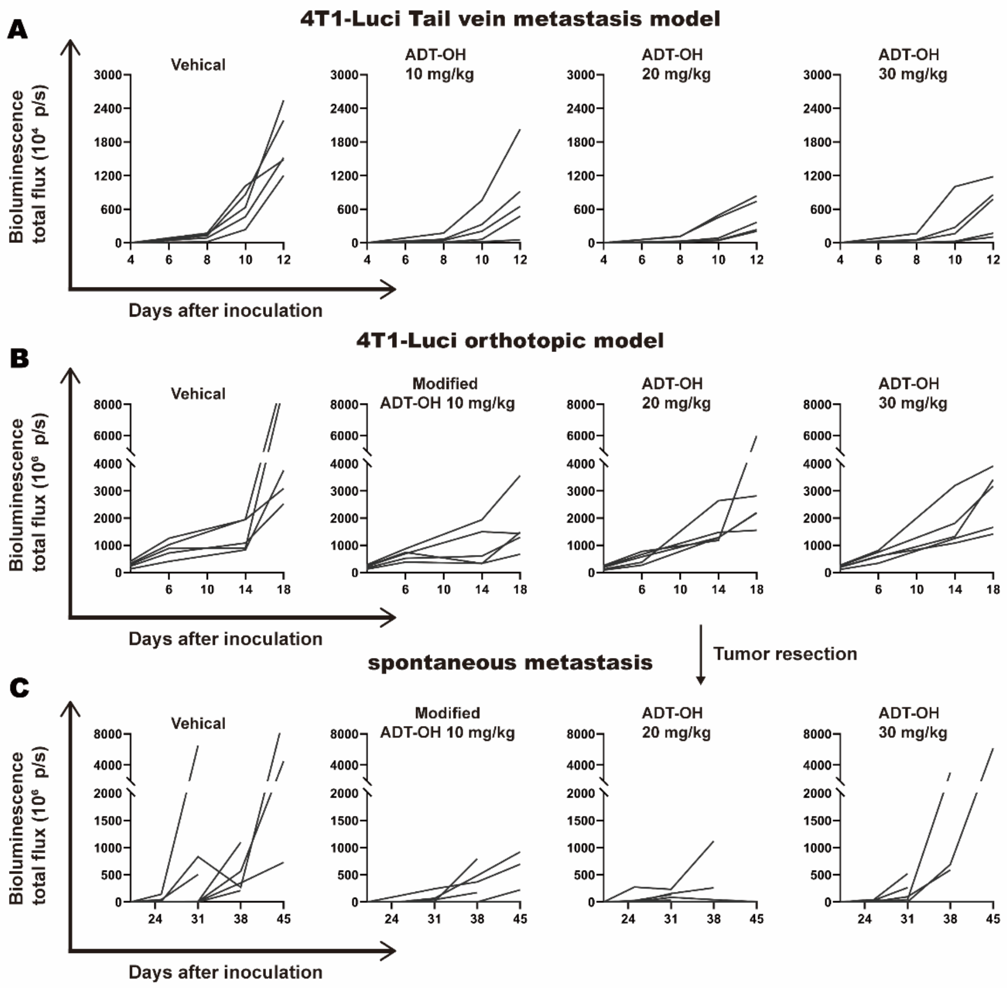

Supplementary Fig. 11 Tumor bioluminescence intensity growth kinetics in different treatment groups of 4T1-Luci syngeneic models

(A) Quantitative analysis of bioluminescence images presented in Fig. 8B. **(B-C)** Quantitative analysis of bioluminescence images presented in Supplementary Fig. 10B-C. The curves were stopped when the corresponding mouse died.
