# Supplementary material for: ADT-OH exhibits anti-metastatic activity on triple-negative breast cancer by combinatorial targeting of autophagy and mitochondrial fission
Source: Cell Death Dis. 2024 Jun 28;15(6):463. doi: 10.1038/s41419-024-06829-w (PMC11213877; doi:10.1038/s41419-024-06829-w)
Supplement: Supplementary file 2 — Original Data File [file 41419_2024_6829_MOESM2_ESM.pdf]

Images of the original western blots-Figure 3C

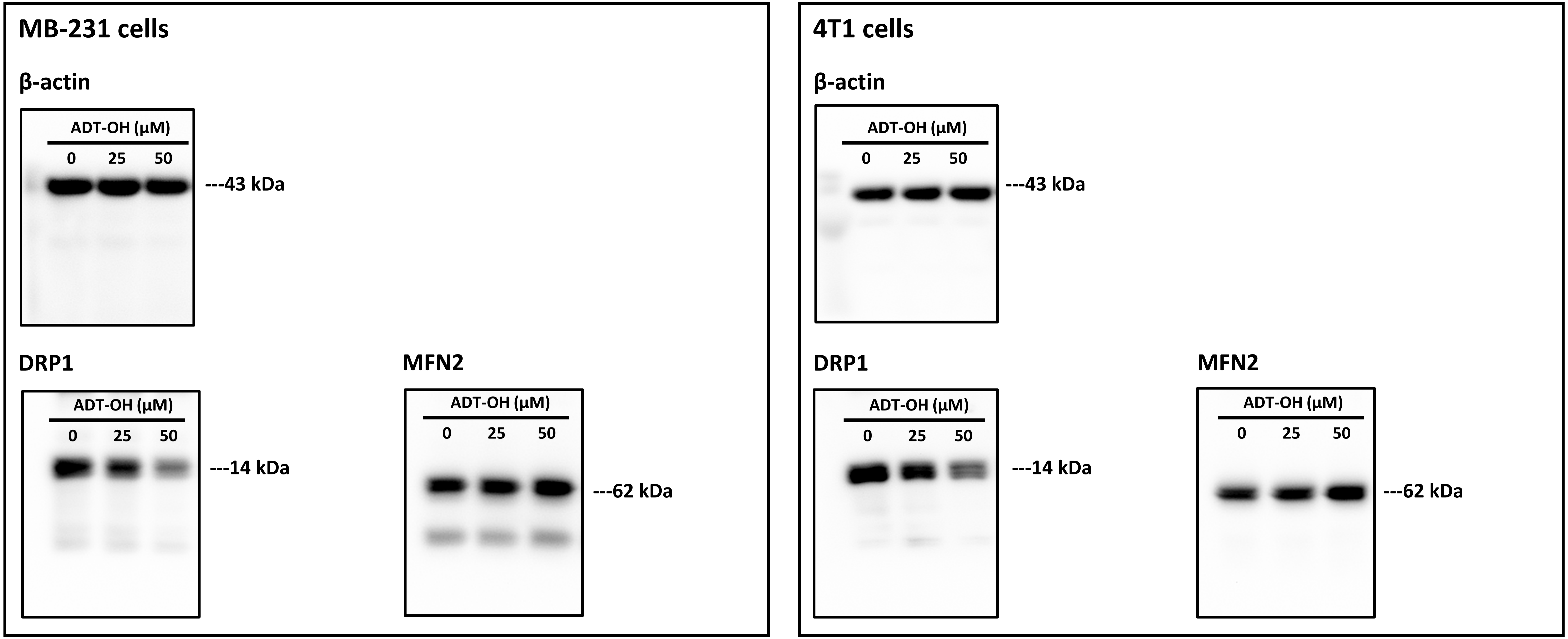

Images of the original western blots-Figure 3C

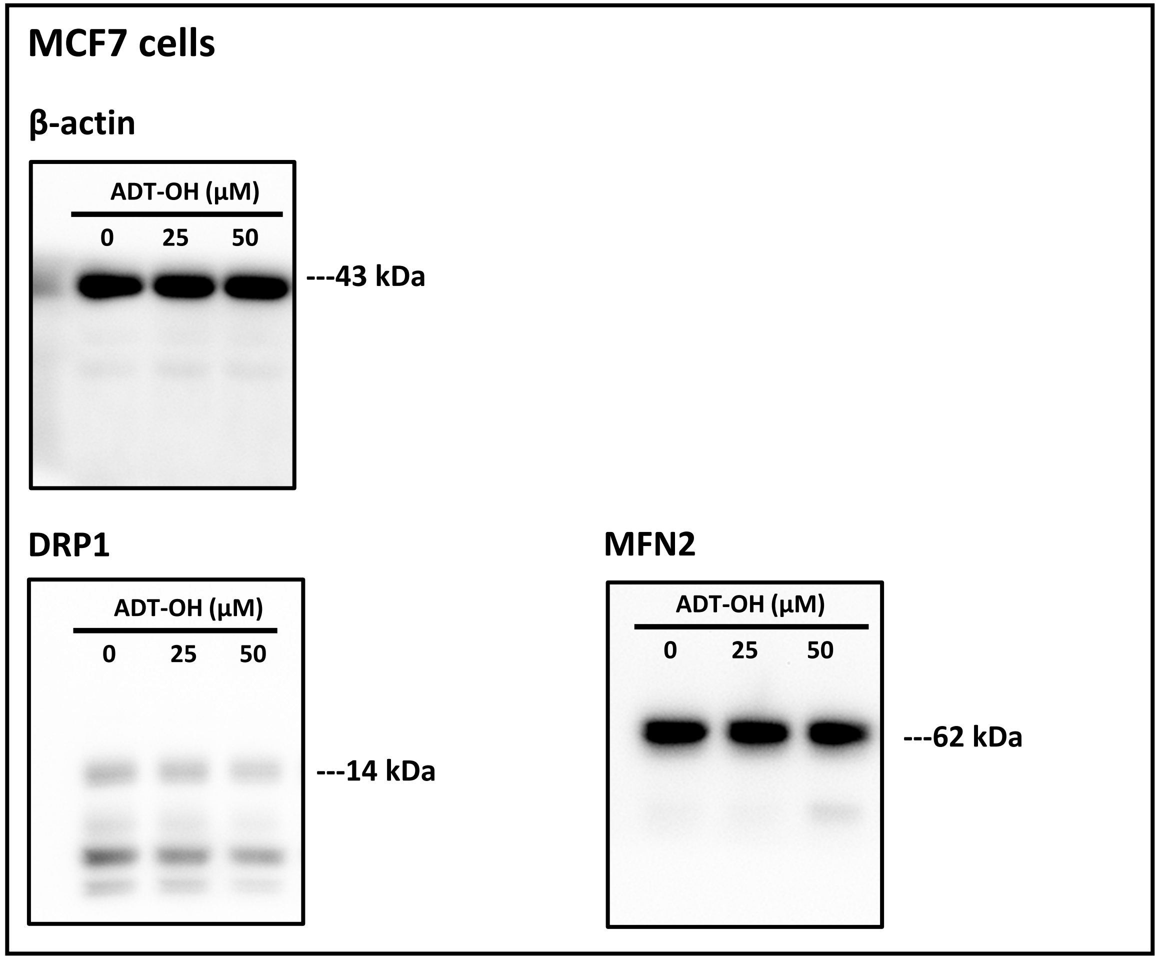

Images of the original western blots-Figure 5A

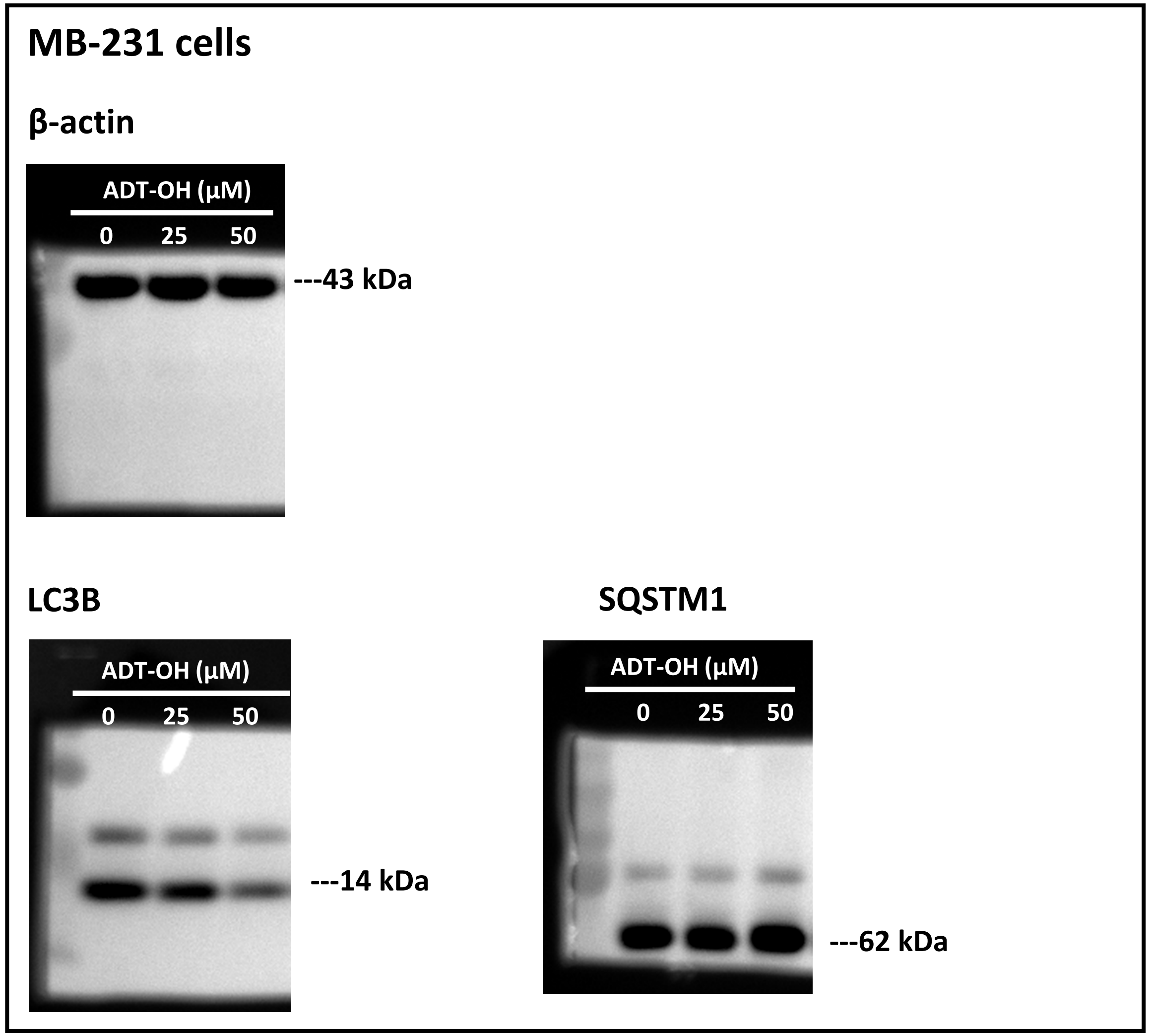

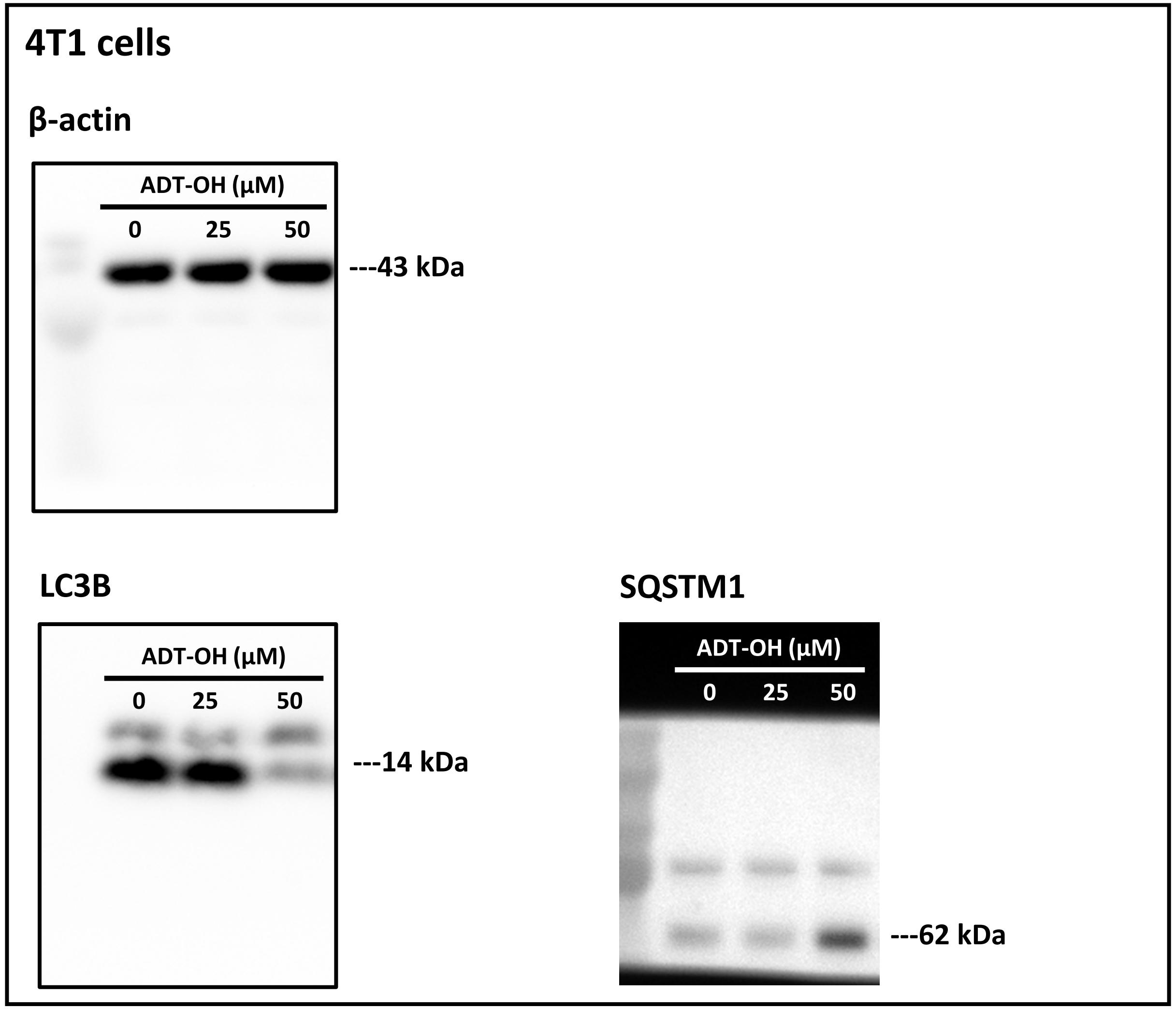

Images of the original western blots-Figure 5A

MCF7 cells

β-actin

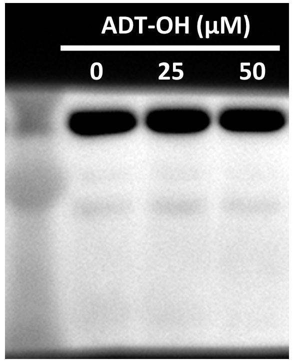

---43 kDa

LC3B

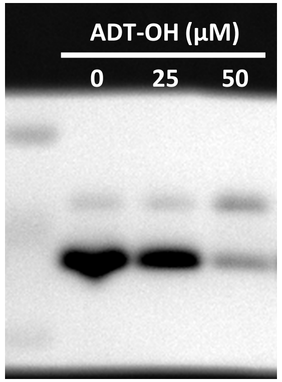

---14 kDa

SQSTM1

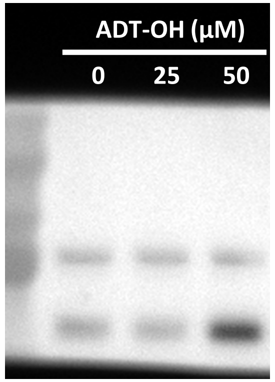

---62 kDa
